# Functional Sites Induce Long-Range Evolutionary Constraints in Enzymes

**DOI:** 10.1371/journal.pbio.1002452

**Published:** 2016-05-03

**Authors:** Benjamin R. Jack, Austin G. Meyer, Julian Echave, Claus O. Wilke

**Affiliations:** 1 Department of Integrative Biology, Center for Computational Biology and Bioinformatics, and Institute for Cellular and Molecular Biology, The University of Texas at Austin, Austin, Texas, United States of America; 2 Escuela de Ciencia y Tecnología, Universidad Nacional de San Martín, San Martín, Buenos Aires, Argentina; Brandeis University, UNITED STATES

## Abstract

Functional residues in proteins tend to be highly conserved over evolutionary time. However, to what extent functional sites impose evolutionary constraints on nearby or even more distant residues is not known. Here, we report pervasive conservation gradients toward catalytic residues in a dataset of 524 distinct enzymes: evolutionary conservation decreases approximately linearly with increasing distance to the nearest catalytic residue in the protein structure. This trend encompasses, on average, 80% of the residues in any enzyme, and it is independent of known structural constraints on protein evolution such as residue packing or solvent accessibility. Further, the trend exists in both monomeric and multimeric enzymes and irrespective of enzyme size and/or location of the active site in the enzyme structure. By contrast, sites in protein–protein interfaces, unlike catalytic residues, are only weakly conserved and induce only minor rate gradients. In aggregate, these observations show that functional sites, and in particular catalytic residues, induce long-range evolutionary constraints in enzymes.

## Abstract 2

Catalytic sites in enzymes are highly conserved, but do they affect the evolutionary conservation of neighboring sites? This study shows that not just nearby neighbors but also second, third, fourth, and even fifth neighbors of a catalytic residue experience evolutionary constraint compared to a random site.

## Author Summary

The basic biochemical functions of life are carried out by large molecules called enzymes. Enzymes consist of long chains of amino acids folded into a three-dimensional structure. Within that structure, a specific cluster of amino acids, known as the active site, performs the biochemical function. Substituting one amino acid for another in the active site typically results in a defective, non-functional enzyme, and therefore mutations at or near enzyme active sites are often lethal. Moreover, even mutations far from the active site have been found to disrupt function. Nonetheless, as organisms evolve, enzymes accumulate random mutations. Where in enzymes’ structures do these mutations accumulate without causing harm? Here, we observe evidence for extensive interactions between active sites and distant regions of the enzyme structure, in a comprehensive set of over 500 enzymes. We show that active sites tightly control the substitutions that an enzyme can tolerate. This control extends far beyond regions of the enzyme immediately adjacent to the active site, covering over 80% of a typical enzyme structure. Our findings have broad implications for molecular evolution, for enzyme engineering, and for the computational prediction of active-site locations in novel enzymes.

## Introduction

Enzymes facilitate the chemical reactions necessary for life. To function properly, enzymes must reconcile two competing demands: they must fold stably into the correct three-dimensional conformation, and they must display the correct catalytic residues in their active sites. As enzymes evolve, mutations that are functionally beneficial are often deleterious for stability, and vice versa [1–3]. Thus, the patterns of evolutionary divergence observed in enzyme evolution are shaped by the interplay of these two potentially conflicting constraints.

Mutations affecting fold stability can occur anywhere in the protein structure, though in general stability effects tend to be more pronounced in the interior, more densely packed regions of a structure than on the protein surface [4,5]. By contrast, where mutations affect function in a protein structure is less clear. Site-directed mutagenesis experiments demonstrate that mutations at catalytic residues, unsurprisingly, disable enzyme function [6,7]. Accordingly, residues directly involved in protein function tend to be more conserved over evolutionary time than other residues [8–10]. Less intuitively, however, mutations 20 Å or more from a catalytic residue can attenuate catalytic activity in enzymes such as glycosidase [11], TEM-lactamase [12], or copper nitrate reductase [13]. Similarly, a study of a small set of α/β-barrel enzymes has found that evolutionary conservation decays continuously with the distance to the nearest catalytic residue [14]. These results suggest that residues far from an active site may be functionally important, but that this importance may decline with distance in physical, three-dimensional space.

Here, we analyze a dataset of 524 distinct enzyme structures spanning the six major functional classes of enzymes. We systematically assess how site-specific evolutionary variation in these enzymes relates to the geometric location of residues relative to the nearest catalytic residue. We find that, across all six major classes of enzymes, the constraining effects of catalytic residues extend to most of an enzyme’s structure, irrespective of protein size. These effects exist regardless of whether an active site is located on the surface or in the core of a protein, and they remain even when controlling for other structural features predicting evolutionary variation. Finally, we find that we can use site-specific conservation gradients to accurately recover active sites in more than 50% of enzymes. In summary, these findings demonstrate that functional sites induce long-range evolutionary constraints in enzyme structures.

## Results

### Functional Sites Induce Gradients of Conservation

To systematically explore the relationship between site-specific evolutionary rates and distance to the nearest catalytic residue, we have analyzed 524 diverse enzyme structures. We have chosen these structures as a subset of enzymes analyzed previously for their relationship between protein structure and evolutionary variation [15]. The structures represent all six major classes of enzymes, and no two structures in the dataset share more than 25% of their respective amino-acid sequences. The dataset includes both single subunit proteins (monomers) and multi-subunit proteins (multimers), and annotations describing the biological assembly and the location of the catalytic residues are available for each structure (see Methods for details). For each enzyme, we have constructed alignments of up to 300 homologous sequences, selected from the UniRef90 database [16,17]. We estimate evolutionary variation at each site in each alignment by calculating a site-specific relative evolutionary rate, using the software Rate4Site [18]. The relative rates are normalized such that a value of one corresponds to the average rate in a given protein, and larger or smaller values represent proportionally larger or smaller rates. For brevity, we will also refer to the relative rates simply as “rates.” In mathematical expressions, rates will be denoted by the letter *K*.

**Fig 1:**
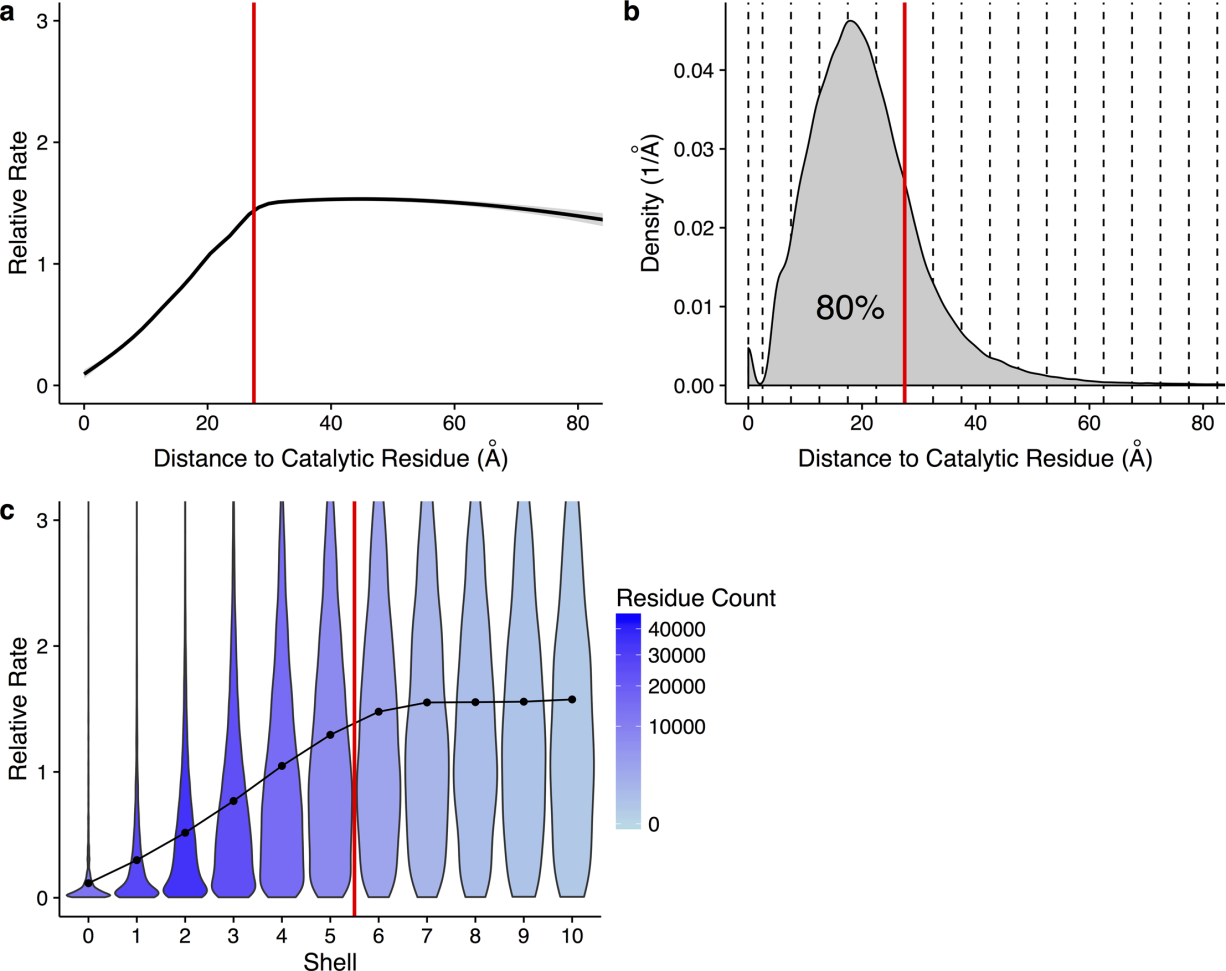
Site-specific evolutionary rates increase with distance to a catalytic residue in 80% of residues. (a) Site-specific evolutionary rate versus distance to the nearest catalytic residue. The curve represents the mean rate for all residues, as calculated from a generalized additive model. Standard error is shown in light gray. Individual residues are not shown. The vertical line indicates a distance of 27.5 Å. On average, rates increase almost linearly with distance, up to ∼27.5 Å. (b) Density plot of residues with a given distance to a catalytic residue. Dashed lines indicate the boundaries of shells with a thickness of 5 Å each. The vertical red line again shows a distance of 27.5 Å, and 80% of residues in the dataset fall within this distance. (c) Distribution of site-specific evolutionary rates within each shell. Violins show the distribution of rates in each shell, points represent the mean, and the shading represents the number of residues found in that shell. The red line indicates a distance of 27.5 Å, corresponding to the boundary of shell 5. When visualizing rate trends with discrete shells, rates increase from shells 0 through 6. Data underlying this figure are available on Github: https://github.com/benjaminjack/enzyme_distance/tree/master/figure_data.

We first ask whether there is an overall trend toward increased evolutionary conservation near active sites. To address this question, we pool all sites from all structures into one combined dataset and then calculate the mean evolutionary rate as a function of Euclidean distance to the nearest catalytic residue in the respective structure. As expected, we find that evolutionary rates are, on average, the lowest at or directly near catalytically active sites. Moreover, we find that rates increase approximately linearly with increasing distance to the nearest catalytic residue, up to a distance of approximately 27.5 Å (Fig 1A). Beyond this distance, rates level off. Importantly, 80% of all residues in our dataset fall within a distance of 27.5 Å to the nearest catalytic residue (Fig 1B). Thus, the vast majority of all residues in each protein appear to experience some amount of purifying selection mediated by catalytic residues.

We can think of sites in a protein as organized into shells according to their distance to the closest catalytic residue. Each shell is 5 Å in width, the approximate minimum distance between two amino acid side-chains. The boundaries between these discrete shells are indicated in Fig 1B with dashed lines, and we can see clear dips in the distribution at 2.5 Å and 7.5 Å, the boundaries between the 0th and 1st and the 1st and 2nd shells (the boundaries between shells become less precise for higher shell numbers). We can subdivide the sites of our dataset into these discrete shells and then plot the rate distribution within each shell (Fig 1C). We find that the mean rate for each shell increases up to shell 6 (32.5 Å) and then stabilizes. Similarly, the width of the distribution also increases up to shell 6. Thus, all shells include some proportion of conserved sites, but increasingly distant shells include an increasing fraction of moderately or highly variable sites.

### Conservation Gradients Are Distinct from Known Structural Constraints on Protein Evolution

The broad rate distributions that we observe within individual shells, in particular within shells distant from catalytic residues, highlight that there are other factors besides distance that also influence the extent and type of selection acting on individual sites. In fact, one important evolutionary constraint is the requirement for proteins to fold stably into their active conformation [19]. This constraint causes sites in the interior of the protein, shielded from the solvent and involved in many inter-residue contacts, to be more evolutionarily conserved than sites on the protein surface (for a recent review, see [30]) [4,15,20–29].

Two structural measures are commonly used to quantify this structural constraint: relative solvent accessibility (RSA) [31] and weighted contact number (WCN) [32]. RSA measures the exposure of a given residue to a hypothetical small solvent molecule, typically water. RSA is useful for determining if a residue is on the surface or the interior of a protein structure. WCN measures the local packing density of a given residue. WCN is high in the core of the protein, where residues are tightly packed. We have calculated both WCN and RSA for each site in each protein in our dataset. We have based this calculation on the published biological assembly of each protein, so that intra-chain contacts are properly accounted for in the case of enzymes that natively function in a multimeric state. As has been reported previously, on average WCN displays higher correlations with site-specific rate than RSA does, in particular when WCN is calculated with respect to the side-chain coordinates of each residue (see also S1 Fig) [33]. However, in our dataset, correlations of rate with WCN are only moderately higher than correlations with RSA, and there are proteins for which RSA outperforms WCN (S1 Fig). Therefore, throughout this work, we consider both WCN and RSA as measures of structural constraints acting on site-specific protein evolution. Importantly, neither WCN nor RSA make any assumptions about catalytic residues in proteins. Both quantities are purely geometric measures of protein structure. Conversely, the distance *d* to the closest catalytic residue does not explicitly contain information about packing density or solvent accessibility. Yet, in our dataset, the three quantities WCN, RSA, and *d* are all correlated with each other (S2 Fig). Therefore, we next ask to what extent the distance *d* captures an evolutionary constraint that is distinct from the constraints captured by WCN and RSA.

**Fig 2:**
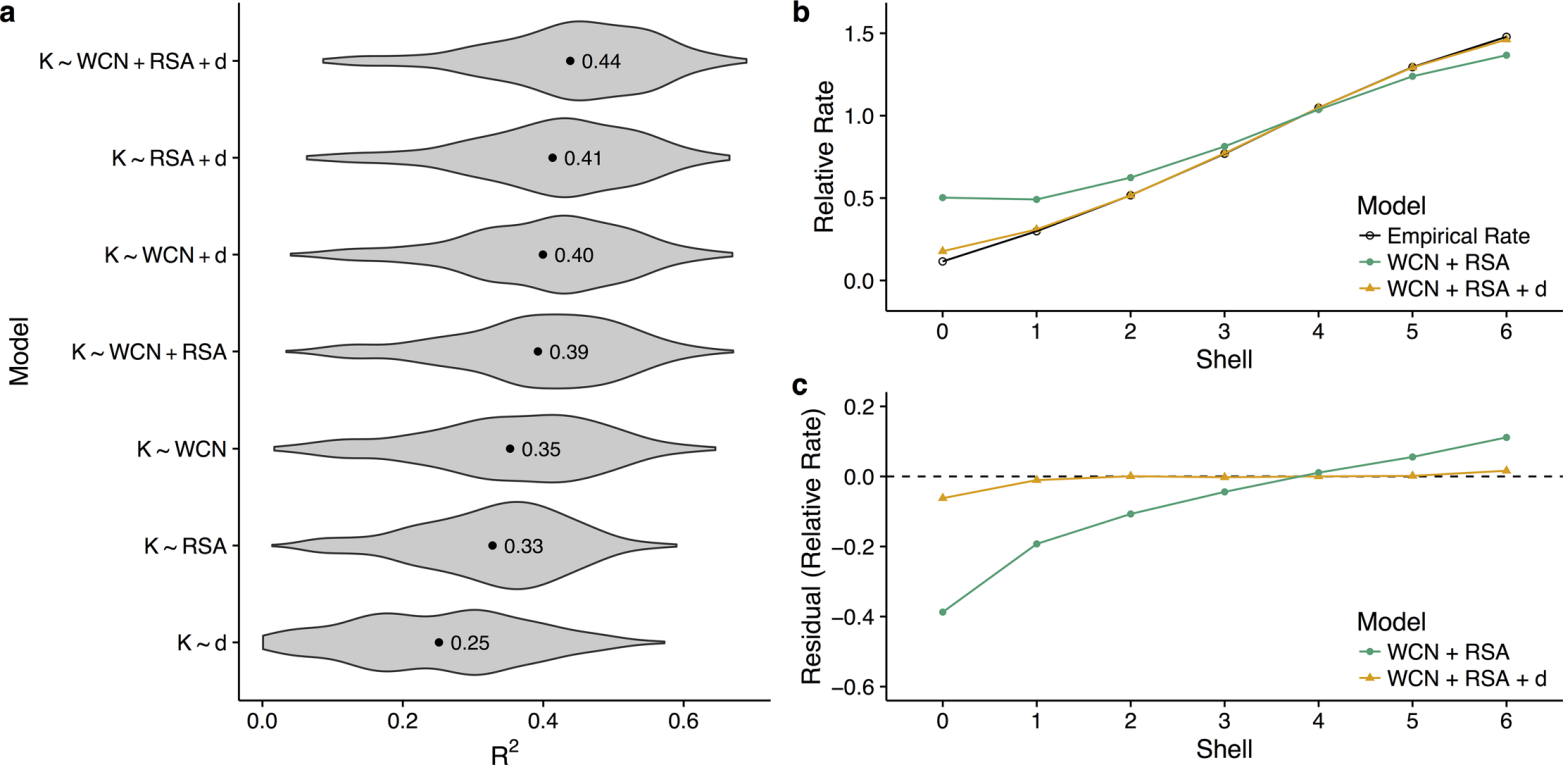
Relative performance of functional and structural predictors of rate. (a) Distribution of *R*^2^ values for various linear models explaining rate, fitted individually for each enzyme. Labeled points within each distribution indicate the mean *R*^2^ value across enzymes. For each enzyme, we regress site-specific evolutionary rate (*K*) against some combination of the distance to the nearest catalytic residue (*d*), weighted contact number (WCN), and relative solvent accessibility (RSA). On average, the addition of distance to the structural constraints WCN and RSA increases the percent variance explained by the linear models by at least 5 percentage points. (b) Mean empirical and predicted rates, separated by shell. A model containing only WCN and RSA overestimates rates near the active site of the enzyme, and the addition of distance corrects this behavior. (c) Mean residuals for linear models with and without distance as a parameter, separated by shell. Without distance, WCN and RSA cannot accurately predict rates within shells 0–4, or equivalently within a distance of 17.5 Å from a catalytic residue. See S3 Fig for plots of relative rate and residuals versus shell for additional models. Data underlying this figure are available on Github: https://github.com/benjaminjack/enzyme_distance/tree/master/figure_data.

To address this question, we regress site-specific evolutionary rates *K* against WCN, RSA, and *d*, in all possible combinations, and separately for each enzyme in our dataset. We then record the *R*^2^ for each model and each enzyme (Fig 2A). We find that the best purely structural model, using both WCN and RSA as predictor variables, explains on average 39% of the variation in rate (Fig 2A). Adding distance as a third predictor to this model increases the average *R*^2^ to 44%. Thus, distance explains on average at least 5% of rate variation that cannot be attributed to purely structural factors, and possibly more than that; by itself, distance explains on average 25% of the variation in rate. Some of that variation may be accidentally captured by WCN or RSA, because active sites are frequently located closer to the interior than to the surface of the protein structure.

To further assess the independent contribution of distance to the pattern of site-specific rates, we compare model predictions and empirical rates as functions of distance to the active site. We compare rates predicted by the linear models *K* ∼ WCN + RSA and *K* ∼ WCN + RSA + *d*, which are fit for each protein individually. For visualization only, we average within shells, as explained above. We find that a linear model containing only WCN and RSA tends to overestimate site-specific evolutionary rates within the first three to four shells (green line in Fig 2B and 2C). Adding distance to this model removes nearly all of the overestimation (orange line in Fig 2B and 2C). These findings demonstrate that structural metrics alone are unable to accurately predict conservation patterns near active sites.

### Functional and Structural Constraints Align for Active Sites in the Interior But Not for Those on the Surface

Enzymes often sequester substrates into a buried catalytic core. This sequestration allows them to facilitate chemistry that would otherwise be impossible in the broader cellular environment. For this reason, many enzymes tend to have active sites in the protein interior, where local packing density is high and solvent accessibility is low [19]. For those enzymes, we expect the distance metric *d* to correlate with WCN and/or RSA. By contrast, if the active site is located on the protein surface, then distance should correlate very little or not at all with either WCN or RSA.

**Fig 3:**
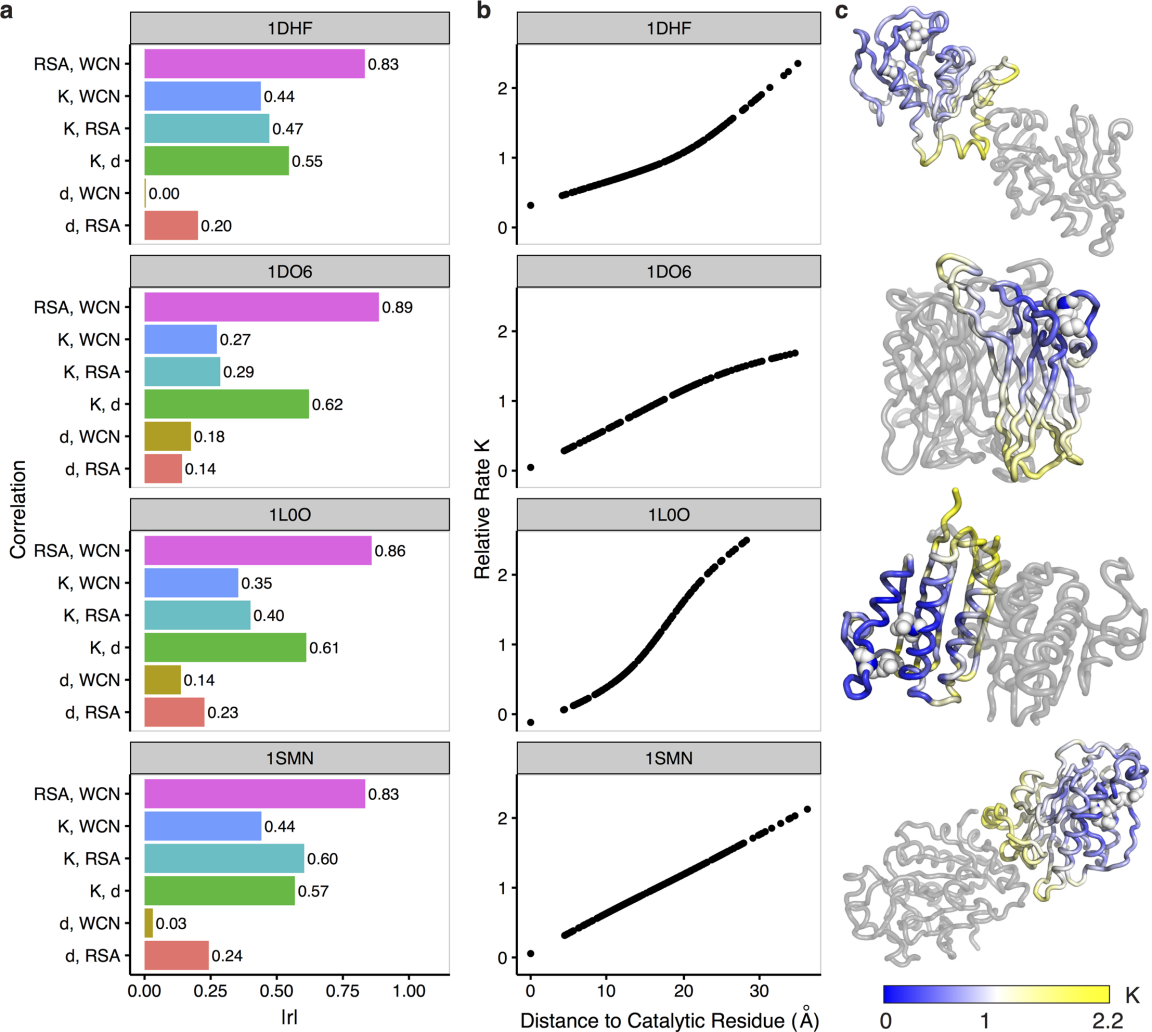
Example enzymes in which distance *d* to the nearest catalytic residue does not correlate strongly with either WCN or RSA. From top to bottom, the PDB IDs of the enzyme structures shown are 1DHF, 1DO6, 1L0O, and 1SMN. (a) Absolute Pearson correlations *r* between combinations of distance *d*, weighted contact number (WCN), relative solvent accessibility (RSA), and rate *K*. Distance correlates weakly with WCN or RSA and strongly with rate, indicating that distance is a strong predictor independent of structural constraints RSA and WCN. (b) Rates smoothed with a GAM (generalized additive model) as a function of *d*, shown for every residue in each enzyme structure. Smoothed rates increase with distance throughout the entire enzyme structure. (c) Enzyme structures colored by smoothed rate *K*. Gray structures represent other subunits in the biological assembly. Active sites in these enzymes (indicated by white spheres) are located outside of the core and away from the interface of subunits. Data underlying this figure are available on Github: https://github.com/benjaminjack/enzyme_distance/tree/master/figure_data.

To further disentangle active-site effects from WCN and RSA, we can identify individual structures from our dataset in which distance is sufficiently uncorrelated (defined as *r* < 0.25) from both WCN and RSA. Among these structures, we find four for which distance correlates strongly with evolutionary rate (defined as *r* ≥ 0.55) (see Fig 3). They correspond to the enzymes dihydrofolate reductase (DHFR, protein databank identifier [PDB ID]: 1DHF) [34], superoxide reductase (SOR, PDB ID: 1DO6) [35], anti-sigma factor SpollAB (PDB ID: 1L0O) [36], and the *Serratia* endonuclease (PDB ID: 1SMN) [37]. All of these enzymes perform different biological functions, and they are active in multimeric conformations. In these proteins, rate correlates more strongly with distance than it does with WCN or RSA (Fig 3A), and the mean rate increases linearly with distance throughout the entire structure (Fig 3B). In all four cases shown, the active sites are located near the protein surface (mean active-site RSA ranges from 0.19 to 0.25) and away from the protein center (Fig 3C).

To analyze the effect of active-site location on rate variation more systematically, we next subdivide our entire dataset into three categories based on active site location, measured by the mean RSA of all catalytic residues in the structure. We define these categories as active site in the protein interior (mean catalytic-residue RSA < 0.05), active site with intermediate solvent exposure (mean catalytic-residue RSA between 0.05 and 0.25), and active site on the protein surface (mean catalytic-residue RSA ≥ 0.25). Our dataset contains 98, 367, and 59 proteins in these three categories, respectively.

**Fig 4:**
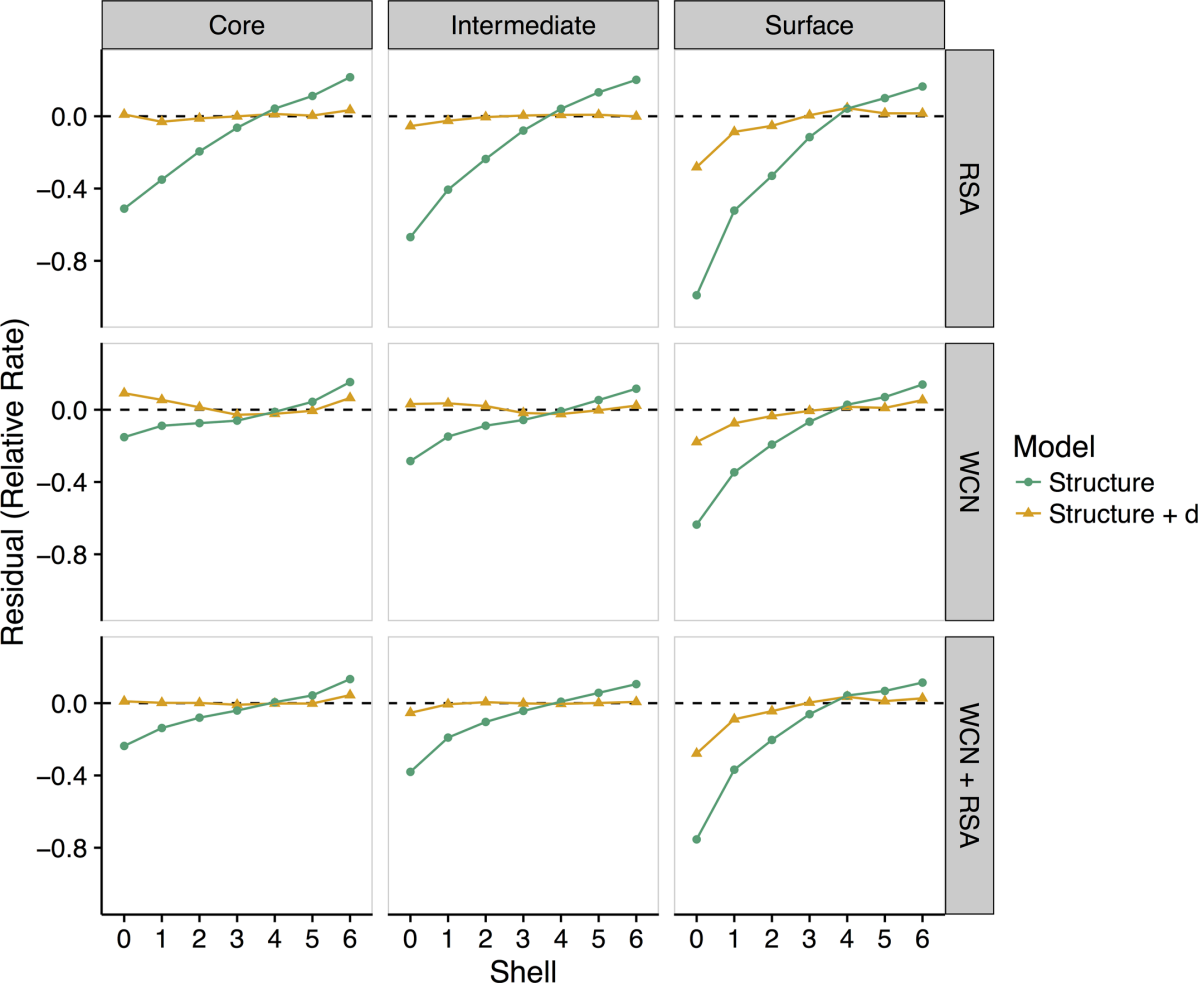
Effect of active-site location on the relationship between site-specific evolutionary rates and distance to the nearest catalytic residue. Enzymes are grouped into three categories, based on the mean solvent exposure of their catalytic residues: core (RSA < 0.05), intermediate (0.05 < RSA < 0.25), and surface (RSA > 0.25). Lines represent mean residuals for different structural linear models (vertical labels, right) and those same models with distance added as a variable. As the active site location moves from the core to the surface of an enzyme, structural constraints (WCN, RSA, and WCN + RSA) increasingly overestimate rates within shells 3–4. Models including distance as a variable predict rates more accurately in shells 0–6, even when the active site is located on the surface. See S4 Fig for plots of residuals versus shell for an additional model, *K* ~ *d*. Data underlying this figure are available on Github: https://github.com/benjaminjack/enzyme_distance/tree/master/figure_data.

As before, we find that the purely structural metrics RSA and WCN tend to overestimate site-specific evolutionary rates near the active site in all three groups (Fig 4). Moreover, the structure-based models perform worse as the active site moves from the core of the enzyme to the surface. In all cases, incorporating distance into the model corrects most rate overestimation near the active site. Interestingly, all models perform better for active sites in the core than for active sites on the surface (Fig 4). We interpret this observation as follows: When the active site is located in the core of an enzyme, functional and structural constraints are aligned. The sites most conserved due to function are also the sites most conserved due to structure, and this overall trend is captured well in the linear models. By contrast, when the active site is located on the surface, functional and structural constraints are at odds with each other. The sites most conserved due to function are now the sites least conserved due to structure, and vice versa. In this case, since there are now two opposing trends within one structure, it is more difficult for any linear model to accurately capture rate variation throughout the structure.

### Distance Effect Extends Further in Larger Proteins

We have previously seen that approximately 80% of all residues in our dataset fall within the 27.5 Å cutoff, inside of which evolutionary variation is reduced in proportion to distance to the nearest active site (Fig 1B). However, the 80% figure may be somewhat misleading, because in that analysis we have pooled all residues from all proteins. Our dataset comprises proteins of very different sizes, from 95 to 1,287 amino acids long, and for small proteins every residue falls within 27.5 Å of an active site, while for large proteins only one-half to two-thirds of the residues lie within the 27.5 Å distance cutoff.

**Fig 5:**
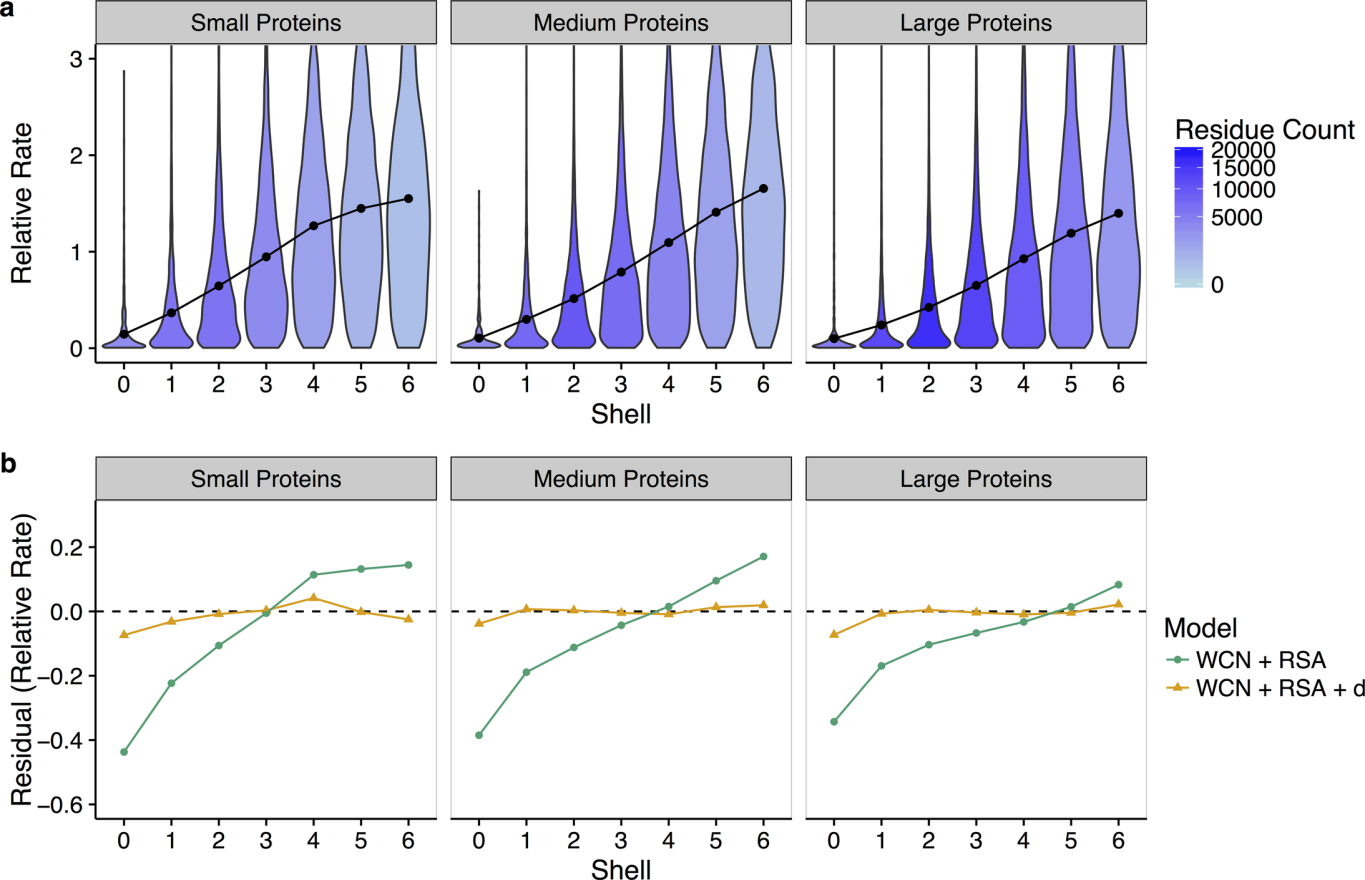
Effect of protein size on the relationship between site-specific evolutionary rates and distance to the nearest catalytic residue. (a) Distribution of rates within each shell, where proteins have been separated into small (95–368 sites), medium (270–385 sites), and large (386–1,287 sites) based on amino-acid sequence length. Points represent the mean rate in each shell. Shading denotes the quantity of residues in each shell. As the size of the protein increases, the distance–rate slope decreases and distance effects extend further from the active site. (b) Mean residuals in each shell for models with and without distance, again separated by protein size. The addition of distance to a structural-constraints (RSA and WCN) model increases the accuracy of rate prediction near the active site up to shell 3 (17.5 Å) in small proteins and up to shell 5 (27.5 Å) in large proteins. Thus, the constraining effects of catalytic residues depend on protein size, with stronger, more local effects in small proteins, and weaker, longer-range effects in large proteins. See S5 Fig for plots of residuals versus shell for an additional model, *K ~ d*. Data underlying this figure are available on Github: https://github.com/benjaminjack/enzyme_distance/tree/master/figure_data.

To ascertain whether the relationship between functional sites and evolutionary rates depends on enzyme size, we can re-analyze our data by protein size. We define three evenly sized groups: small proteins (95–268 sites), medium-size proteins (270–385 sites), and large proteins (386–1,287 sites). Each group contains 175, 175, and 174 structures, respectively. We observe that as enzyme size increases, the rate–distance slope decreases (Fig 5A). Distance effects are weaker in larger proteins but also extend further out. The effect remains visible when we analyze the distance–rate relationship for individual proteins and in the context of WCN and RSA (Figs 5B and S5): purely structural models, which use only WCN and RSA to predict rate, overestimate rate up to shell 3 in small proteins, up to shell 4 in medium-sized proteins, and up to shell 5 in larger proteins.

In summary, we see that the leveling off of rate at shell 5, around 27.5 Å, in the pooled dataset, does not represent a universal cutoff but rather an average obtained from combining many different structures into one analysis. For any individual protein, there will generally be a distance effect, but it may extend only to shell 3 or 4 in small proteins while extending to shell 6 (and possibly beyond) for very large proteins.

### Selection Gradients Recover Catalytic Residues

As we have seen from the preceding analyses, active sites in enzymes impose a selection gradient that can be detected throughout the majority of the protein structure. This observation leads us to ask whether we can use this gradient to identify active sites when their location is not known. To answer this question, we blindly search for distance–rate gradients in our dataset. We systematically use one residue at a time as a reference point in the structure and fit a linear model of rate versus the distance to that reference point. We record the resulting *R*^2^ for each model, and we consider the reference point with the highest *R*^2^ as the putative active site in the structure.

**Fig 6:**
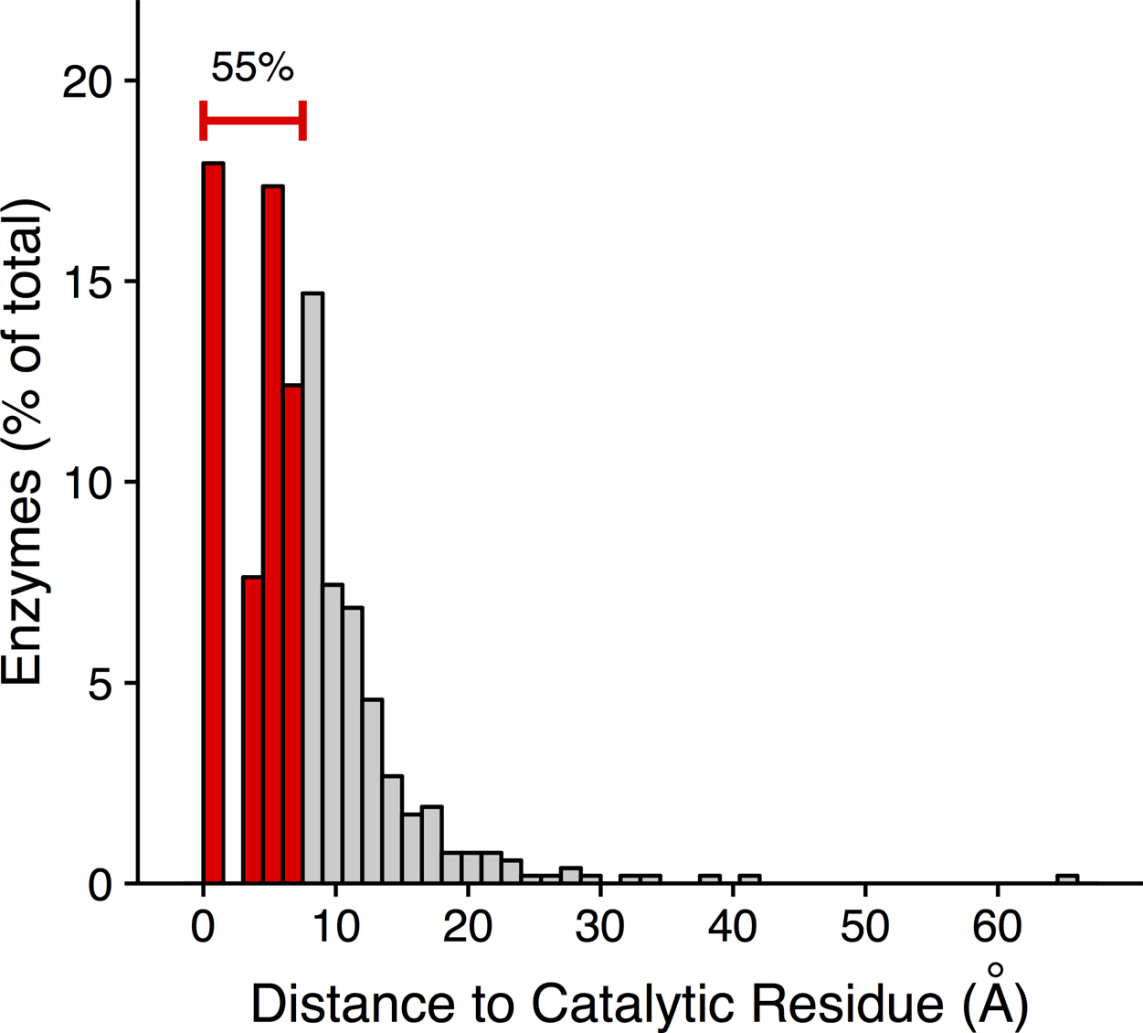
Catalytic residues can be recovered from site-specific evolutionary-rate gradients. To determine the putative catalytic residue in an enzyme, we first select each residue in the structure, one at a time, as a reference residue. Then we regress rate against the set of distances from the reference residue. The putative catalytic residue is the reference residue that maximizes the model’s *R*^2^. We have plotted here the distribution of distances of these putative catalytic residues to the nearest known catalytic residue. A distance of zero indicates that the putative catalytic residue is a true catalytic residue. 18% of enzymes have a putative catalytic residue that is a true catalytic residue. An additional 37% of enzymes have a putative catalytic residue within 7.5 Å (shell 1) of a catalytic residue. In total, we recover a catalytic residue or a residue in immediate contact with a catalytic residue in 55% of the enzymes. Data underlying this figure are available on Github: https://github.com/benjaminjack/enzyme_distance/tree/master/figure_data.

We find that in 18% of the structures in our dataset, the putative active site coincides with a known catalytic residue (Fig 6). In an additional 37% of structures, the putative active site falls within 7.5 Å of a catalytic residue but is not a catalytic residue itself. A distance of 7.5 Å corresponds to one shell, i.e., it captures residues in direct contact with a catalytic residue. Note that the gap visible between 0 and 2.5 Å in Fig 6 corresponds to the closest distance that two side chains can physically contact each other. A putative active site either is a catalytic residue, in which case it has a distance of 0 Å to the nearest catalytic residue (i.e., itself), or alternatively it has to be at least a distance of 2.5 Å away from the catalytic residue. In summary, for more than half (55%) of the 524 enzymes in our dataset, we can use the existing selection gradient to identify either a catalytic residue or an immediate neighbor.

As a control, we have also considered a model that places the active site at the core of the protein, at the residue with the overall highest WCN (since, as stated above, the active site is located in the protein interior for many enzymes). We find that this control approach recovers catalytic residues or their immediate neighbors in 31% of enzymes (S6 Fig). Thus, while the control approach can recover active sites in a substantial fraction of enzymes, the selection-gradient-based method performs significantly better (odds ratio = 2.8, *p* < 1.7 x 10^−15^, Fisher’s Exact Test, S7 Fig, S1 Table).

### Catalytic Residues Evolve More Slowly Than Protein–Protein Interface Residues

**Fig 7:**
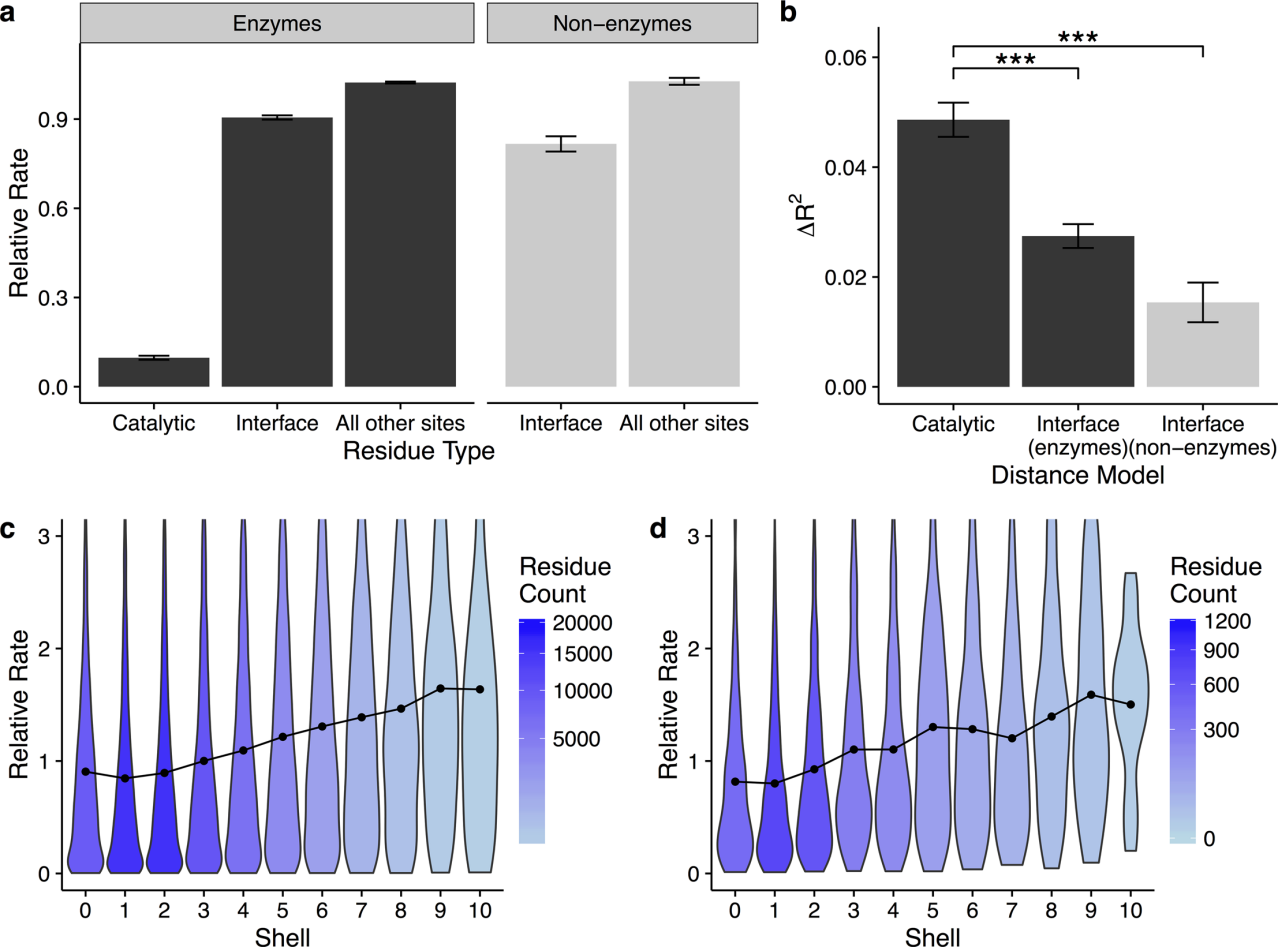
Catalytic residues are more conserved than residues in protein–protein interfaces, and interface residues induce much weaker evolutionary rate gradients relative to catalytic residues. (a) Mean evolutionary rates of catalytic residues and of interface residues. Interface residues are only slightly more conserved than non-interface residues in both the enzyme (*p* < 2.2 x 10^−16^, *t* test) and non-enzyme (*p* < 1.5 x 10^−13^, *t* test) datasets. Catalytic residues, by comparison, are highly conserved relative to all other residues (*p* < 2.2 x 10^−16^ for both pairwise comparisons, *t* test). (b) Change in variance explained by the addition of distance to structural models that predict rates. Distance to a catalytic residue improves predictions of rate more than the addition of distance to interface residues (*** indicates *p* < 3.8 x 10^−8^, *t* test). (c–d) Distribution of rates within each shell, where shells correspond to the distance to the nearest interface residue in (c) enzymes and (d) non-enzymes. Points represent the mean rate in each shell. Shading denotes the quantity of residues in each shell. In both enzymes and non-enzymes, the evolutionary rate gradients relative to interface residues are weaker than gradients relative to catalytic residues. Data underlying this figure are available on Github: https://github.com/benjaminjack/enzyme_distance/tree/master/figure_data.

Many enzymes function as components of multimeric protein complexes. In fact, more than half of the enzymes in our dataset contain multiple subunits in their biological assemblies. The arrangement of and interaction between these subunits could substantially modify how protein structure and protein function shape protein evolution, especially if the active site occurs at the interface of two subunits. In our dataset, we find that residues in protein–protein interfaces are, on average, only slightly more conserved than any other residues, whereas catalytic residues are much more conserved (Fig 7A): residues in interfaces evolve, on average, at a rate of 0.91 relative to the average residue, while catalytic residues evolve, on average, at a relative rate of 0.10. To verify that the little conservation we see in interface sites is not an artifact of our enzyme dataset, we have also analyzed rates in a set of 17 non-enzymatic protein–protein complexes, consisting of 30 individual proteins total (see Methods). Again, we find that residues in protein–protein interfaces show only moderate conservation relative to all other residues (relative rate of 0.82, Fig 7A). Moreover, consistent with their weak conservation, protein–protein interfaces induce only very minor gradients of conservation, if any, in both enzyme and non-enzyme proteins (compare Fig 7B–7D with Fig 1). Thus, protein–protein interactions impose much weaker evolutionary constraints than catalytic sites.

The structural metrics RSA and WCN are also sensitive to subunit arrangement, and subunit arrangement can be incorrectly annotated in the biological assembly. To assess whether subunit arrangement and/or annotation errors affect the distance–rate relationship, we re-analyze our enzyme data using three additional variations of analysis choices: (i) RSA and WCN are calculated using the biological assembly, and any residues at the interface between subunits are excluded; (ii) RSA and WCN are calculated using a single subunit, and no residues are excluded; and (iii) RSA and WCN are calculated using a single subunit, and all interface residues are excluded (S8–S34 Figs). In all three cases, our results remain qualitatively unchanged from our prior results: rate increases with increasing distance to a catalytic residue, up to about 27.5 Å; distance has an effect on rate variation that is independent from the purely structural metrics WCN and RSA. Thus, in summary, there is a positive distance–rate relationship that is independent of WCN or RSA, and it exists regardless of how we treat multi-subunit enzymes and interface residues.

## Discussion

We have shown that many enzymes exhibit a clear, nearly linear relationship between site-specific evolutionary rates and distance to the nearest catalytic residue. We have found this trend consistently throughout a large dataset of 524 diverse enzymes, and we have found that the relationship extends to most of the residues in any given enzyme structure. Using combined linear models containing RSA, WCN, and distance, we have found that distance explains at least 5% of the variance in rate after controlling for WCN and RSA, and potentially up to approximately 36% (Fig 3) in proteins in which the active site is located near the protein surface. Moreover, models containing only the structural predictors WCN and RSA consistently overestimate evolutionary variation near active sites, through shell 5 (27.5 Å) in large proteins. Finally, we have shown that in over half of the enzymes in our dataset, we can recover catalytic residues or their immediate neighbors from the evolutionary gradients they imprint throughout the protein structure.

For some enzymes in our dataset, we have found little evidence for functional or structural constraints on site-specific evolutionary rates. There are some proteins for which less than 10% of the variation in evolutionary rate can be accounted for with distance to a catalytic residue, RSA, or WCN. These low correlations suggest that either the rates themselves are uninformative, or that the available PDB structures are not reflective of protein structure in vivo. In the first case, the sequence alignments used to determine evolutionary rate could contain a mix of proteins with very different arrangements in vivo. We have no way of determining the biological assembly of every sequence in the alignment, so differences in corresponding subunit arrangement could bias the site-specific evolutionary rates. Additionally, the RCSB protein database may have conflicting biological assemblies. For example, the biological assembly for human dihydrofolate reductase (PDB ID: 1DHF) is classified as a homodimer, while the biological assembly for a separate structure (PDB ID: 1DRF) of the same protein is classified as a monomer. We have attempted to control for possible structural variability in the sequence alignments and biological assemblies by re-analyzing all structures as monomers and/or removing residues at the interface of subunits before computing correlations, and the overall trends observed remain the same (S8–S34 Figs). Regardless, all of the factors mentioned here could result in rates that correlate poorly with any structural predictors.

The field of molecular evolution has long sought to understand the relationship between protein structure, function, and sequence evolution [30]. Here, we have assessed this relationship by comparing distance to the nearest catalytic residue with site-specific evolutionary rates. Past work has employed covariation analyses to reveal clusters of co-evolving residues in protein structures, deemed “protein sectors” [38]. In specific cases, such as in serine proteases, these protein sectors also correspond to different functional biochemical regions of the structure. A recent reanalysis of the seminal protein sector work demonstrates that, in proteins with just one sector, sequence conservation recovers clusters of functional residues just as well as covariation analyses [39]. Our work demonstrates that not only are clusters of functional residues highly conserved, but that such residues induce gradients of conservation within a structure. This finding of long-range interactions between residues is consistent with the sector model of large regions of co-evolving residues.

We have found that active sites are among the most highly conserved sites in proteins, whereas residues involved in protein–protein interactions are only weakly conserved relative to the average site in a protein. Moreover, the gradients of conservation induced by protein–protein interfaces are much less marked than those induced by catalytic sites. This finding is consistent with prior work on protein–protein interactions. While several prior works have found increased conservation in interface regions [40–42], effect sizes have generally been found to be small. For example, [42] found that the reduction in evolutionary rate in a protein–protein interface was mostly (though not entirely) explained by the reduction in solvent accessibility induced by complex formation. Also, complementation assays and computer simulations suggest that protein–protein interfaces can experience extensive divergence without loss of function [43], again supporting the notion that protein–protein interfaces are frequently not under strong purifying selection. For these reasons, we believe that the rate gradients we have found toward active sites are not strongly confounded by protein–protein binding interfaces.

Long-range interactions between residues in a protein have historically been studied in the context of allostery. Initially proposed in 1961, allostery describes the process by which a small molecule (ligand) binds to one area of an enzyme (allosteric site) and induces a conformational change at a distant active site [44]. Studies of allosteric interactions shed light on two key aspects of our findings. First, biophysical models have been developed that explain long-range interactions. The Monod-Wyman-Changuex model, the most widely studied of allostery models, proposes that ligand binding stabilizes a biologically active or inactive quarternary structure [44]. Recent studies, however, demonstrate that some monomeric proteins also contain allosteric sites [44]. In G-protein coupled receptors, for example, a simplified model of conserved, physically connected amino-acid residues explains the long-range interactions between allosteric sites and active sites [45]. Our dataset contains a mix of monomeric and multimeric proteins, and we observe long-range interactions in both types of proteins. Thus, our findings suggest that allosteric-like couplings between active sites and distant residues may be more common than previously thought. The physical distance between allosteric ligand-binding sites and active sites ranges from 20 Å in hemoglobin to 60 Å in glycogen phosphorylase [46]. Therefore, the selection gradients we have observed here extend to distances well within the range of experimentally observed allosteric interactions. Second, while the observed selection gradients have allowed us to recover residues in close proximity to the active site in a little over half of the proteins in our dataset, in many proteins (45%) the selection gradient points toward a residue >7.5 Å from the active site. It is possible that these non-catalytic residues, which are highly predictive of the overall patterns of evolutionary rates in the structure, may be allosteric sites. Allosteric sites tend to be highly conserved, although typically not as conserved as active sites [47]. In summary, studies of allostery provide biophysical explanations for long-range interactions between residues and may explain why we failed to recover catalytic residues from selection gradients in some proteins in our dataset.

That selection gradients can recover active sites has potentially broad applications, even beyond enzymatic proteins. For example, some of us have previously used optimized distance to identify important functional sites in influenza A hemagglutinin (HA) [48]. HA, a viral surface protein, interacts directly with sialic acid found on the surface of human cells. Viral infection requires binding of HA to sialic acid, and antibodies bind near the sialic-acid binding region to inhibit viral infection. Residues in that region are thus under strong positive selection for immune escape, and consequently the selection gradient in HA revealed a rapidly evolving functional site. This finding suggests that selection gradients could effectively recover diverse types of functional sites, not only those that are well conserved. More broadly, evolutionary history is a useful predictor of active sites [8,9] and binding partners [10]. Assuming that a given structure has been crystalized, the rate gradients we have found here could improve computational predictions of active sites and binding sites.

## Methods

### Datasets and Site-Specific Evolutionary Rates

We selected 524 of 554 previously characterized enzymes [15] to conduct our analysis. We removed 30 structures because they contained chains with no available catalytic residue information, or because the UniRef90 database did not contain enough homologous sequences to construct a diverse alignment. These enzymes consist of 204 monomers and 320 multimers, and no two enzymes in the dataset have more than 25% sequence identity. For each enzyme, we obtained catalytic residue information from the Catalytic Site Atlas [49].

We acquired PDB structures of the biological assemblies for these proteins from the RCSB protein database [50]. A biological assembly represents the functional form of a given enzyme in vivo based on the best experimental data available. When available, we used biological assemblies that are author-provided or both author-provided and software-supported (labeled “A” and “A+S,” respectively, in the RCSB protein database). If author-provided biological assemblies were not available, we used biological assemblies predicted by PISA (protein interfaces, surfaces, and assemblies, http://www.ebi.ac.uk/pdbe/pisa/) (labeled “S”). PISA biological assemblies are entirely predicted by software. In cases in which there were multiple author-provided biological assemblies, we chose the first of those assemblies listed in the RCSB protein database.

In addition to the enzyme dataset, we also compiled a non-enzyme dataset as a control. We selected 17 of 179 protein–protein complexes from the Protein–Protein Interaction Affinity Database 2.0 [51]. We selected only non-enzymatic proteins based on interaction classification, absence of enzyme comission (EC) number, and UniProt annotations. We also excluded complexes containing antibodies, since antibodies evolve on a different time-scale and by different mechanisms than other cellular proteins. We acquired structures of the protein–protein complexes from the RCSB protein database.

To calculate site-specific evolutionary rates, we first extracted the amino-acid sequences from the PDB structures. Using PSI-BLAST [52], we then queried the UniRef90 database [16,17] to retrieve homologous sequences for each enzyme. Among these homologous sequences for each enzyme, we removed sequences with less than 10% pairwise divergence to any other sequence, to reduce phylogenetic bias. Next, we randomly downsampled the homologous sequences to a maximum of 300 sequences per enzyme. Then, we performed a multiple sequence alignment (MSA) of the sequences with MAFFT 7.215 (Multiple Alignment using Fast Fourier Transform) [53] and generated phylogenetic trees with RAxML 7.2.8 (Randomized Axelerated Maximum Likelihood) [54] using the LG substitution matrix (named after Le and Gacuel) [55] and the PROTCAT model of rate heterogeneity [56]. We calculated site-specific evolutionary rates with the program Rate4Site 2.01 [18], using the MSAs and phylogenetic trees from the previous step as input. We used the empirical Bayes approach for rate estimation and the JTT (Jones, Taylor, and Thorton) model of amino acid replacement [57]. Lastly, we normalized the rates such that the rates for each protein have a mean of 1. Because these rates are measured relative to the average divergence rate of the entire protein, they are dimensionless. Throughout this work, we refer to these site-specific relative rates as *K*, or simply “rates.”

### Predictor Variables

For each protein structure, we calculated several predictor variables at each site. First, we calculated the weighted contact number WCN for each residue *i* as follows:

$${\text{WCN}}_{i}=\sum_{j\neq i}\frac{1}{r_{ij}^{2}}.$$ (1)

Here, *r*_*ij*_ is the distance between the geometric center of the side-chain atoms in residue *i* and the geometric center of side-chain atoms in residue *j*. To calculate these distances for residue pairs involving glycine, which has no side-chain, we used the location of the C_α_ in those residues instead. Unless noted otherwise, WCN was calculated using the complete biological assembly of the protein.

Next, we calculated the relative solvent accessibility (RSA) at each site. To this end, we first calculated the accessible surface area (ASA) using the software mkdssp [58,59]. We then normalized ASAs by the maximum solvent accessibility for each residue in a Gly-X-Gly tripeptide [31]. Peptide linkages across chains, typically disulfide bridges, were assigned an RSA of zero. Unless noted otherwise, RSA was calculated using the complete biological assembly of the protein.

Finally, we calculated the distance *d* to the nearest catalytic residue for each residue in each structure. Most enzymes have multiple catalytic residues, so we define *d* as distance to the nearest catalytic residue. As was the case for WCN, distances were measured from the geometric center of the side-chain of one residue to the the geometric center of the side-chain of another residue. And in the case of glycines, the position of C_α_ was again used in place of the side-chain geometric center. Any residue with *d* = 0 is therefore a catalytic residue, and conversely, all catalytic residues lie at *d* = 0.

We defined interface residues as residues for which RSA differed by a minimum of 10% when calculated for the full biological assembly or for a single chain. All interface residues were included in the analyses presented in the main body of the text, but we excluded interface residues in the analyses presented in S17–S34 Figs.

### Linear Models

For each enzyme in the dataset, we fit the following linear models (represented in standard R notation): *K* ~ *d*, *K* ~ RSA, *K* ~ WCN, *K* ~ RSA + *d*, *K* ~ WCN + *d*, and *K* ~ RSA + WCN + *d*, where *K* is site-specific evolutionary rate, RSA is relative solvent accessibility, WCN is weighted contact number, and *d* is distance to the nearest catalytic residue.

### Statistical Analyses, Plots, and Data Availability

All statistical analyses were carried out using the R software package [60]. Linear models are fit to each enzyme individually. After fitting the models, data are then binned for visualization purposes. Plots are generated with ggplot2 [61]. All code and data necessary to reproduce our analyses are available in a Github repository at: https://github.com/benjaminjack/enzyme_distance. Processed enzyme data are also provided as S1 Data. Processed data from the non-enzyme dataset are available as S2 Data. Parameter estimates for each linear model fitted to each enzyme are available in S3 Data.

## Supporting Information

S1 Data Complete final dataset for enzyme data. This data file contains all analyzed quantities (such as rate estimates, WCN values, RSA values, etc.) for every residue in the 524 enzymes we analyzed.
(ZIP)

S2 Data Complete final dataset for non-enzyme data. This data file contains all analyzed quantities (such as rate estimates, WCN values, RSA values, etc.) for every residue in the 17 non-enzyme proteins we analyzed.
(ZIP)

S3 Data Regression coefficients for each linear model fitted to each enzyme. For each regression coefficient, we provide the parameter estimate, standard error, *t* statistic, and *p* value.
(ZIP)

S1 Fig Pearson *r* values of side-chain WCN and RSA correlated with site-specific evolutionary rates. The sign of the correlation coefficients for WCN are switched from negative to positive for a simpler comparison with RSA. Each point corresponds to an individual protein, and the numbers refer to the number of proteins above or below the *y* = *x* line. In 346 proteins, WCN was a better predictor of rate. In 178 proteins, RSA was a better predictor of rate. In aggregate, WCN is a better predictor of site-specific evolutionary rate than RSA. Data underlying this figure are available on Github: https://github.com/benjaminjack/enzyme_distance/tree/master/figure_data.
(TIFF)

S2 Fig Distributions of Pearson *r* correlations between pairs of site-specific evolutionary rate predictors on a per protein basis. Predictor pairs include distance and WCN, distance and RSA, and WCN and RSA. WCN and RSA correlate more strongly with each other than either does with distance. Data underlying this figure are available on Github: https://github.com/benjaminjack/enzyme_distance/tree/master/figure_data.
(TIFF)

S3 Fig Empirical and predicted site-specific evolutionary rates and residuals from different combined linear models. Lines represent structural linear models (vertical labels) and those same models with distance added as a variable. A line representing a linear model with distance *d* alone is also included. (a) Each point is the mean predicted rate for a given shell across all residues in the dataset. (b) Each point is the mean residual for a given shell across all residues in the dataset. In all cases, models that include distance as a variable predict rate more accurately than models containing only structural variables, especially near the active site. Data underlying this figure are available on Github: https://github.com/benjaminjack/enzyme_distance/tree/master/figure_data.
(TIFF)

S4 Fig Effect of active-site location on the relationship between site-specific evolutionary rates and distance to the nearest catalytic residue. As in Fig 4, but also showing residuals for a distance *d* only model. Data underlying this figure are available on Github: https://github.com/benjaminjack/enzyme_distance/tree/master/figure_data.
(TIFF)

S5 Fig Mean residuals for different combined linear models used to predict site-specific evolutionary rate. The dataset is divided into small (95–268 sites), medium (270–385 sites), and large (386–1287 sites) proteins. Each point represents the mean predicted rate for all residues in a given shell. Data underlying this figure are available on Github: https://github.com/benjaminjack/enzyme_distance/tree/master/figure_data.
(TIFF)

S6 Fig Distribution of distances between the site with maximum WCN and the nearest catalytic residue. The site with the maximum WCN in a structure is a catalytic residue or a direct contact of a catalytic residue in 31% of enzymes in the dataset. Data underlying this figure are available on Github: https://github.com/benjaminjack/enzyme_distance/tree/master/figure_data.
(TIFF)

S7 Fig ROC curve indicating the performance of models used to predict catalytic residues. In the optimized *d* model, we select a residue as a reference residue and then regress rate against the set of distances to that reference residue. We record the *R*^2^ and then repeat the process until every residue in the structure has been selected as a reference residue. The predicted catalytic residue is then the reference residue that yields a model with the maximum *R*^2^. As a control, we include a maximum WCN model that places the active site at the protein core. The optimized *d* model (AUC = 0.889) outperforms the maximum WCN model (AUC = 0.809). Data underlying this figure are available on Github: https://github.com/benjaminjack/enzyme_distance/tree/master/figure_data.
(TIFF)

S8 Fig Site-specific evolutionary rates increase with distance to a catalytic residue. As in Fig 1, but using single subunits with interface residues included. Data underlying this figure are available on Github: https://github.com/benjaminjack/enzyme_distance/tree/master/figure_data.
(TIFF)

S9 Fig Relative performance of functional and structural predictors of rate. As in Fig 2, but using single subunits with interface residues included. Data underlying this figure are available on Github: https://github.com/benjaminjack/enzyme_distance/tree/master/figure_data.
(TIFF)

S10 Fig Effect of protein size on the relationship between site-specific evolutionary rates and distance to the nearest catalytic residue. As in Fig 5, but using single subunits with interface residues included. Data underlying this figure are available on Github: https://github.com/benjaminjack/enzyme_distance/tree/master/figure_data.
(TIFF)

S11 Fig Effect of active-site location on the relationship between site-specific evolutionary rates and distance to the nearest catalytic residue. As in S4 Fig, but using single subunits with interface residues included. Data underlying this figure are available on Github: https://github.com/benjaminjack/enzyme_distance/tree/master/figure_data.
(TIFF)

S12 Fig Example enzymes from our dataset. From top to bottom, the PDB IDs of the enzymes structures shown are 1DHF, 1DO6, 1L0O, and 1SMN. As in Fig 3, but using single subunits with interface residues included. Data underlying this figure are available on Github: https://github.com/benjaminjack/enzyme_distance/tree/master/figure_data.
(TIFF)

S13 Fig Pearson *r* values of side-chain WCN and RSA correlated with site-specific evolutionary rates. As in S1 Fig, but using single subunits with interface residues included. Data underlying this figure are available on Github: https://github.com/benjaminjack/enzyme_distance/tree/master/figure_data.
(TIFF)

S14 Fig Distributions of Pearson *r* correlations between pairs of site-specific evolutionary rate predictors on a per protein basis. As in S2 Fig, but using single subunits with interface residues included. Data underlying this figure are available on Github: https://github.com/benjaminjack/enzyme_distance/tree/master/figure_data.
(TIFF)

S15 Fig Empirical and predicted site-specific evolutionary rates from different combined linear models. As in S3 Fig, but using single subunits with interface residues included. Data underlying this figure are available on Github: https://github.com/benjaminjack/enzyme_distance/tree/master/figure_data.
(TIFF)

S16 Fig Mean residuals for different combined linear models used to predict site-specific evolutionary rate. As in S5 Fig, but using single subunits with interface residues included. Data underlying this figure are available on Github: https://github.com/benjaminjack/enzyme_distance/tree/master/figure_data.
(TIFF)

S17 Fig Site-specific evolutionary rates increase with distance to a catalytic residue. As in Fig 1, but using single subunits with interface residues removed. Data underlying this figure are available on Github: https://github.com/benjaminjack/enzyme_distance/tree/master/figure_data.
(TIFF)

S18 Fig Relative performance of functional and structural predictors of rate. As in Fig 2, but using single subunits with interface residues removed. Data underlying this figure are available on Github: https://github.com/benjaminjack/enzyme_distance/tree/master/figure_data.
(TIFF)

S19 Fig Effect of protein size on the relationship between site-specific evolutionary rates and distance to the nearest catalytic residue. As in Fig 5, but using single subunits with interface residues removed. Data underlying this figure are available on Github: https://github.com/benjaminjack/enzyme_distance/tree/master/figure_data.
(TIFF)

S20 Fig Effect of active-site location on the relationship between site-specific evolutionary rates and distance to the nearest catalytic residue. As in Fig 4, but using single subunits with interface residues removed. Data underlying this figure are available on Github: https://github.com/benjaminjack/enzyme_distance/tree/master/figure_data.
(TIFF)

S21 Fig Example enzymes from our dataset. From top to bottom, the PDB IDs of the enzyme structures shown are 1DHF, 1DO6, 1L0O, and 1SMN. As in Fig 3, but using single subunits with interface residues removed. Data underlying this figure are available on Github: https://github.com/benjaminjack/enzyme_distance/tree/master/figure_data.
(TIFF)

S22 Fig Pearson *r* values of side-chain WCN and RSA correlated with site-specific evolutionary rates. As in S1 Fig, but using single subunits with interface residues removed. Data underlying this figure are available on Github: https://github.com/benjaminjack/enzyme_distance/tree/master/figure_data.
(TIFF)

S23 Fig Distributions of Pearson *r* correlations between pairs of site-specific evolutionary rate predictors on a per protein basis. As in S2 Fig, but using single subunits with interface residues removed. Data underlying this figure are available on Github: https://github.com/benjaminjack/enzyme_distance/tree/master/figure_data.
(TIFF)

S24 Fig Empirical and predicted site-specific evolutionary rates from different combined linear models. As in S3 Fig, but using single subunits with interface residues removed. Data underlying this figure are available on Github: https://github.com/benjaminjack/enzyme_distance/tree/master/figure_data.
(TIFF)

S25 Fig Mean residuals for different combined linear models used to predict site-specific evolutionary rate. As in S5 Fig, but using single subunits with interface residues removed. Data underlying this figure are available on Github: https://github.com/benjaminjack/enzyme_distance/tree/master/figure_data.
(TIFF)

S26 Fig Site-specific evolutionary rates increase with distance to a catalytic residue. As in Fig 1, but using biological assemblies with interface residues removed. Data underlying this figure are available on Github: https://github.com/benjaminjack/enzyme_distance/tree/master/figure_data.
(TIFF)

S27 Fig Relative performance of functional and structural predictors of rate. As in Fig 2, but using biological assemblies with interface residues removed. Data underlying this figure are available on Github: https://github.com/benjaminjack/enzyme_distance/tree/master/figure_data.
(TIFF)

S28 Fig Effect of protein size on the relationship between site-specific evolutionary rates and distance to the nearest catalytic residue. As in Fig 5, but using biological assemblies with interface residues removed. Data underlying this figure are available on Github: https://github.com/benjaminjack/enzyme_distance/tree/master/figure_data.
(TIFF)

S29 Fig Effect of active-site location on the relationship between site-specific evolutionary rates and distance to the nearest catalytic residue. As in Fig 4, but using biological assemblies with interface residues removed. Data underlying this figure are available on Github: https://github.com/benjaminjack/enzyme_distance/tree/master/figure_data.
(TIFF)

S30 Fig Example enzymes from our dataset. From top to bottom, the PDB IDs of the enzyme structures shown are 1DHF, 1DO6, 1L0O, and 1SMN. As in Fig 3, but using biological assemblies with interface residues removed. Data underlying this figure are available on Github: https://github.com/benjaminjack/enzyme_distance/tree/master/figure_data.
(TIFF)

S31 Fig Pearson *r* values of side-chain WCN and RSA correlated with site-specific evolutionary rates. As in S1 Fig, but using biological assemblies with interface residues removed. Data underlying this figure are available on Github: https://github.com/benjaminjack/enzyme_distance/tree/master/figure_data.
(TIFF)

S32 Fig Distributions of Pearson *r* correlations between pairs of site-specific evolutionary rate predictors on a per protein basis. As in S2 Fig, but using biological assemblies with interface residues removed. Data underlying this figure are available on Github: https://github.com/benjaminjack/enzyme_distance/tree/master/figure_data.
(TIFF)

S33 Fig Empirical and predicted site-specific evolutionary rates from different combined linear models. As in S3 Fig, but using biological assemblies with interface residues removed. Data underlying this figure are available on Github: https://github.com/benjaminjack/enzyme_distance/tree/master/figure_data/.
(TIFF)

S34 Fig Mean residuals for different combined linear models used to predict site-specific evolutionary rate. As in S5 Fig, but using biological assemblies with interface residues removed. Data underlying this figure are available on Github: https://github.com/benjaminjack/enzyme_distance/tree/master/figure_data/.
(TIFF)

S1 Table Comparison of two active-site recovery methods, one based on evolutionary-rate gradients and the other based on strictly structural properties. The “Distance” column refers to the distance between the predicted catalytic residue and a true catalytic residue. “Optimized *d*” refers to selecting a reference residue that yields the maximum *R*^2^ between the set of distances to that residue and rates. This reference residue is predicted to be a catalytic residue. “Max. WCN” refers to a method in which the site with the maximum weighted contact number is assumed to be a catalytic residue. Evolutionary rate gradients recover active sites more often than does the site with maximum packing density (oddsratio = 2.8, *p* < 1.7 x 10^−15^, Fisher’s Exact Test).
(DOCX)

## Abbreviations

ASA: accessible surface area
EC: enzyme comission
MSA: multiple sequence alignment
PDB ID: protein databank identifier
PISA: protein interfaces, surfaces and assemblies
RSA: relative solvent accessibility
WCN: weighted contact number
