# Supplementary figures and images for: Functional Sites Induce Long-Range Evolutionary Constraints in Enzymes
Source: PLoS Biol. 2016 May 3;14(5):e1002452. doi: 10.1371/journal.pbio.1002452 (PMC4854464; doi:10.1371/journal.pbio.1002452)

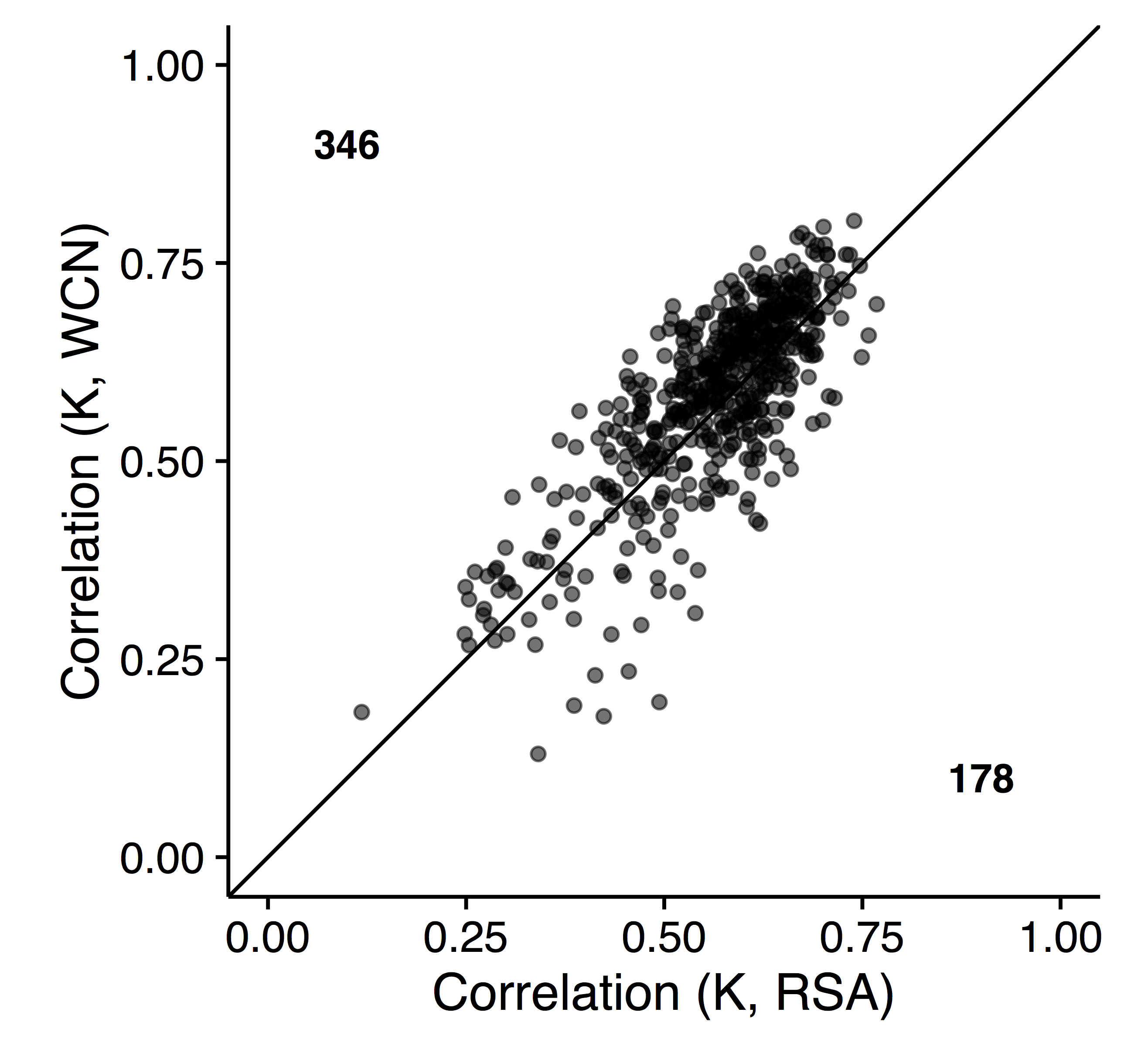

Supplement: S1 Fig — The sign of the correlation coefficients for WCN are switched from negative to positive for a simpler comparison with RSA. Each point corresponds to an individual protein, and the numbers refer to the number of proteins above or below the y = x line. In 346 proteins, WCN was a better predictor of rate. In 178 proteins, RSA was a better predictor of rate. In aggregate, WCN is a better predictor of site-specific evolutionary rate than RSA. Data underlying this figure are available on Github: https://github.com/benjaminjack/enzyme_distance/tree/master/figure_data. (TIFF) [file pbio.1002452.s004.tiff]

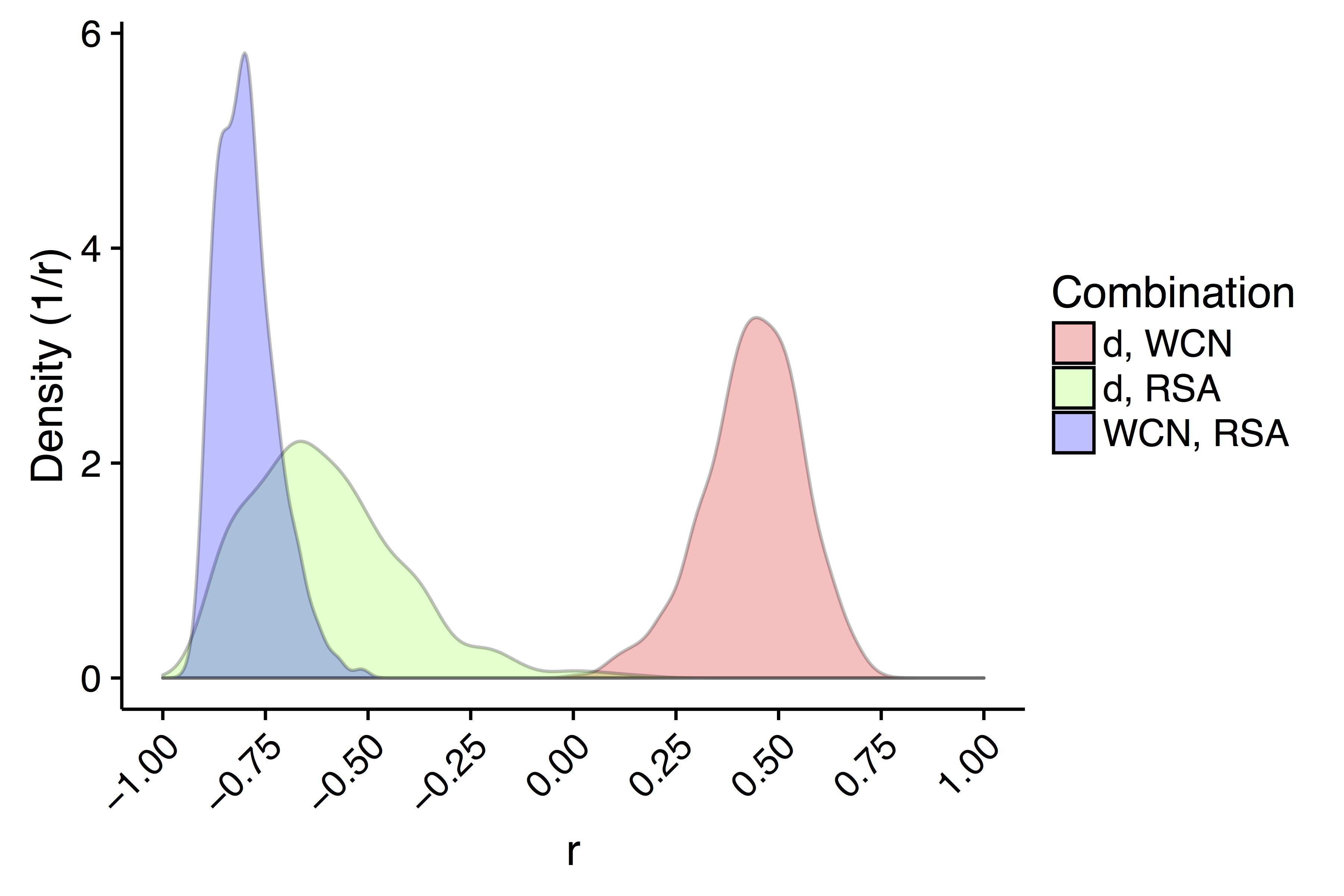

Supplement: S2 Fig — Predictor pairs include distance and WCN, distance and RSA, and WCN and RSA. WCN and RSA correlate more strongly with each other than either does with distance. Data underlying this figure are available on Github: https://github.com/benjaminjack/enzyme_distance/tree/master/figure_data. (TIFF) [file pbio.1002452.s005.tiff]

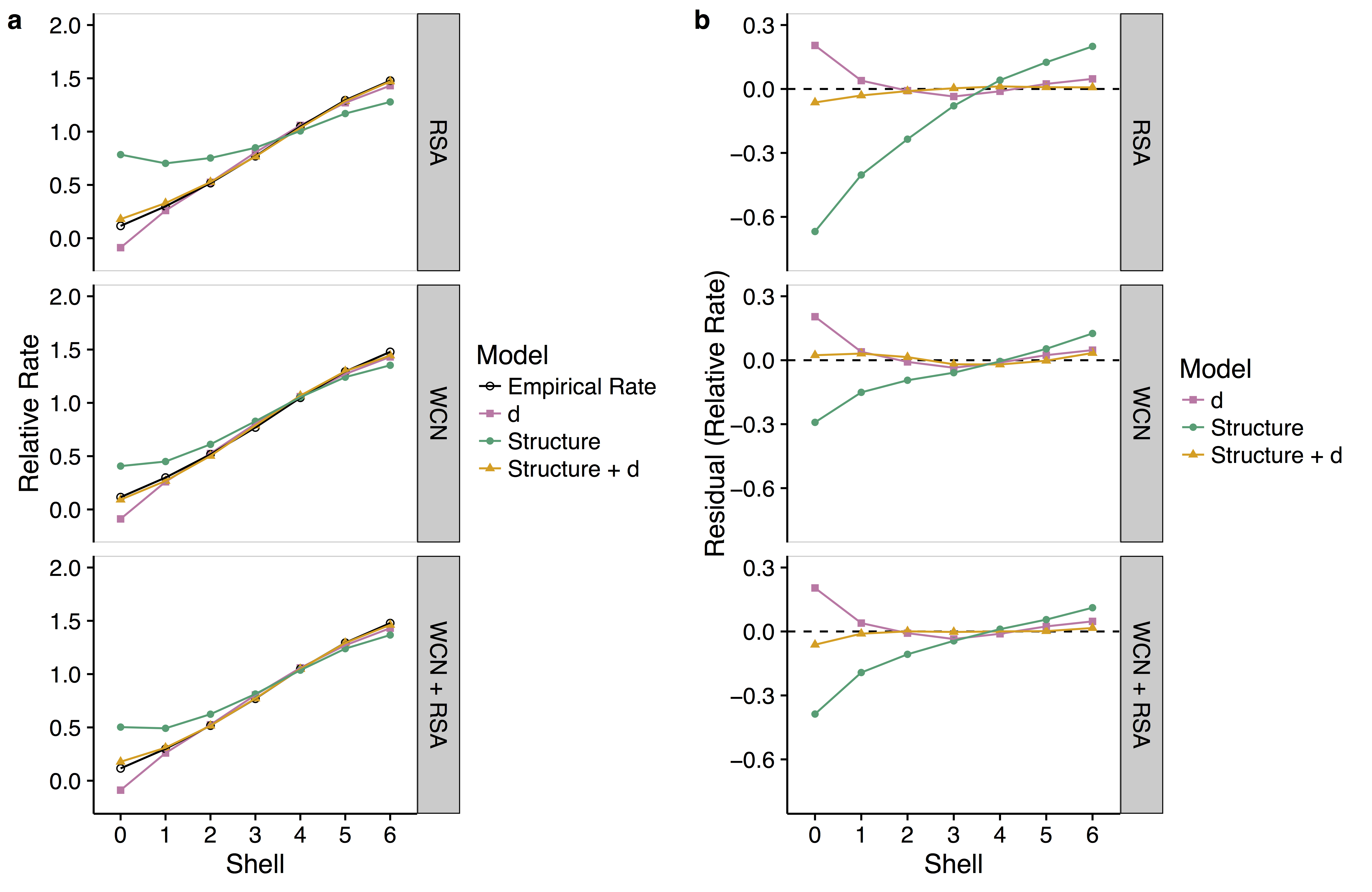

Supplement: S3 Fig — Lines represent structural linear models (vertical labels) and those same models with distance added as a variable. A line representing a linear model with distance d alone is also included. (a) Each point is the mean predicted rate for a given shell across all residues in the dataset. (b) Each point is the mean residual for a given shell across all residues in the dataset. In all cases, models that include distance as a variable predict rate more accurately than models containing only structural variables, especially near the active site. Data underlying this figure are available on Github: https://github.com/benjaminjack/enzyme_distance/tree/master/figure_data. (TIFF) [file pbio.1002452.s006.tiff]

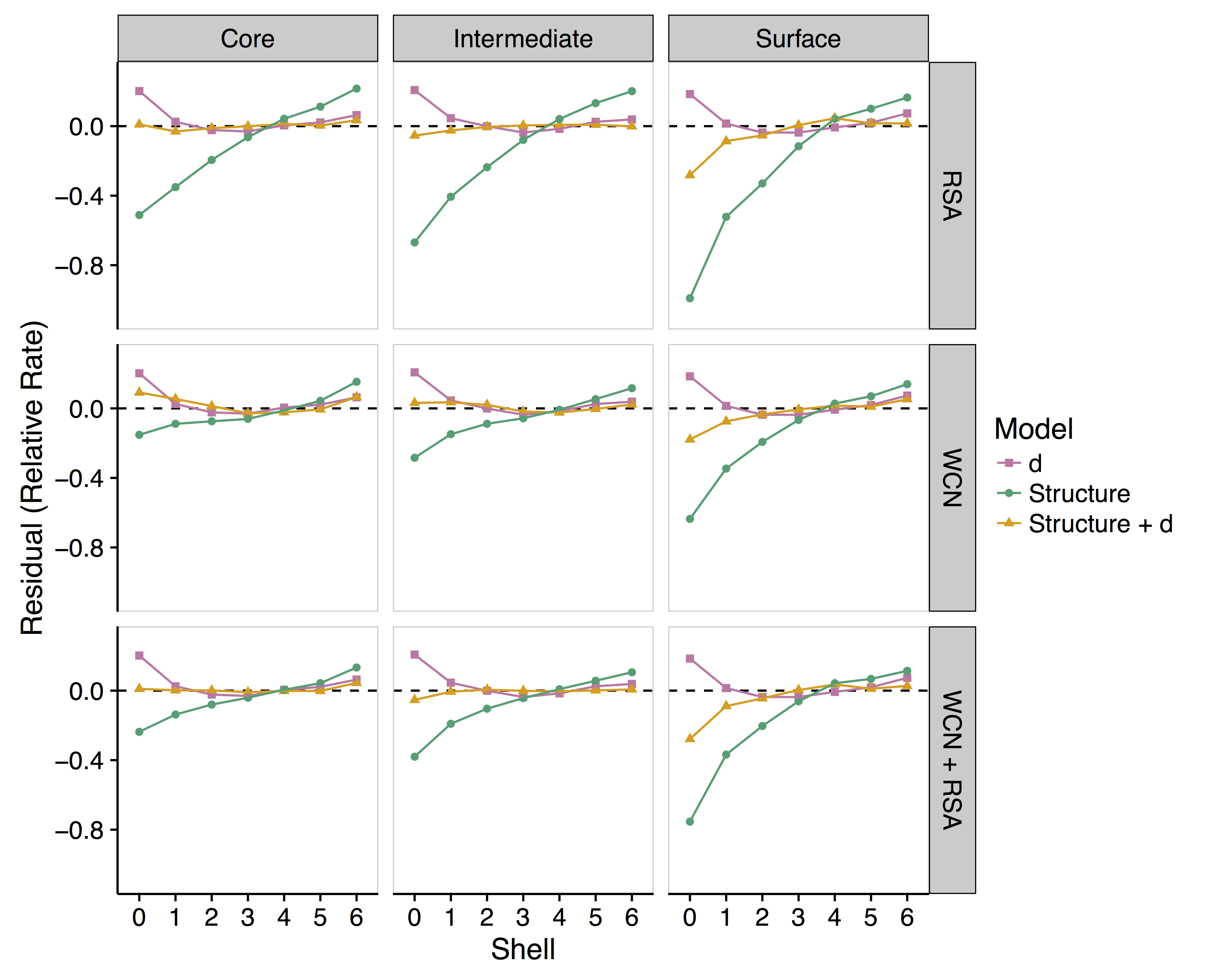

Supplement: S4 Fig — As in Fig 4, but also showing residuals for a distance d only model. Data underlying this figure are available on Github: https://github.com/benjaminjack/enzyme_distance/tree/master/figure_data. (TIFF) [file pbio.1002452.s007.tiff]

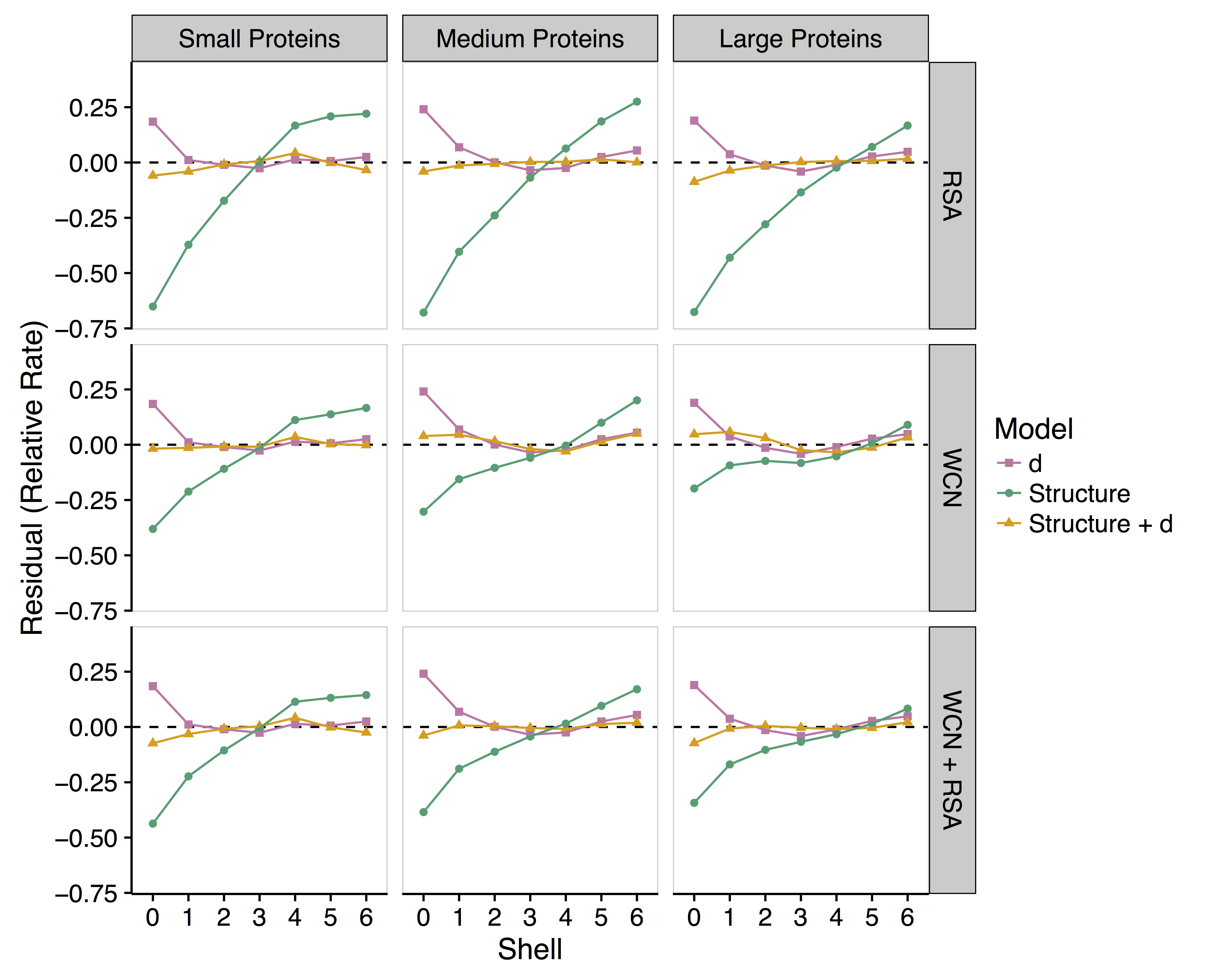

Supplement: S5 Fig — The dataset is divided into small (95–268 sites), medium (270–385 sites), and large (386–1287 sites) proteins. Each point represents the mean predicted rate for all residues in a given shell. Data underlying this figure are available on Github: https://github.com/benjaminjack/enzyme_distance/tree/master/figure_data. (TIFF) [file pbio.1002452.s008.tiff]

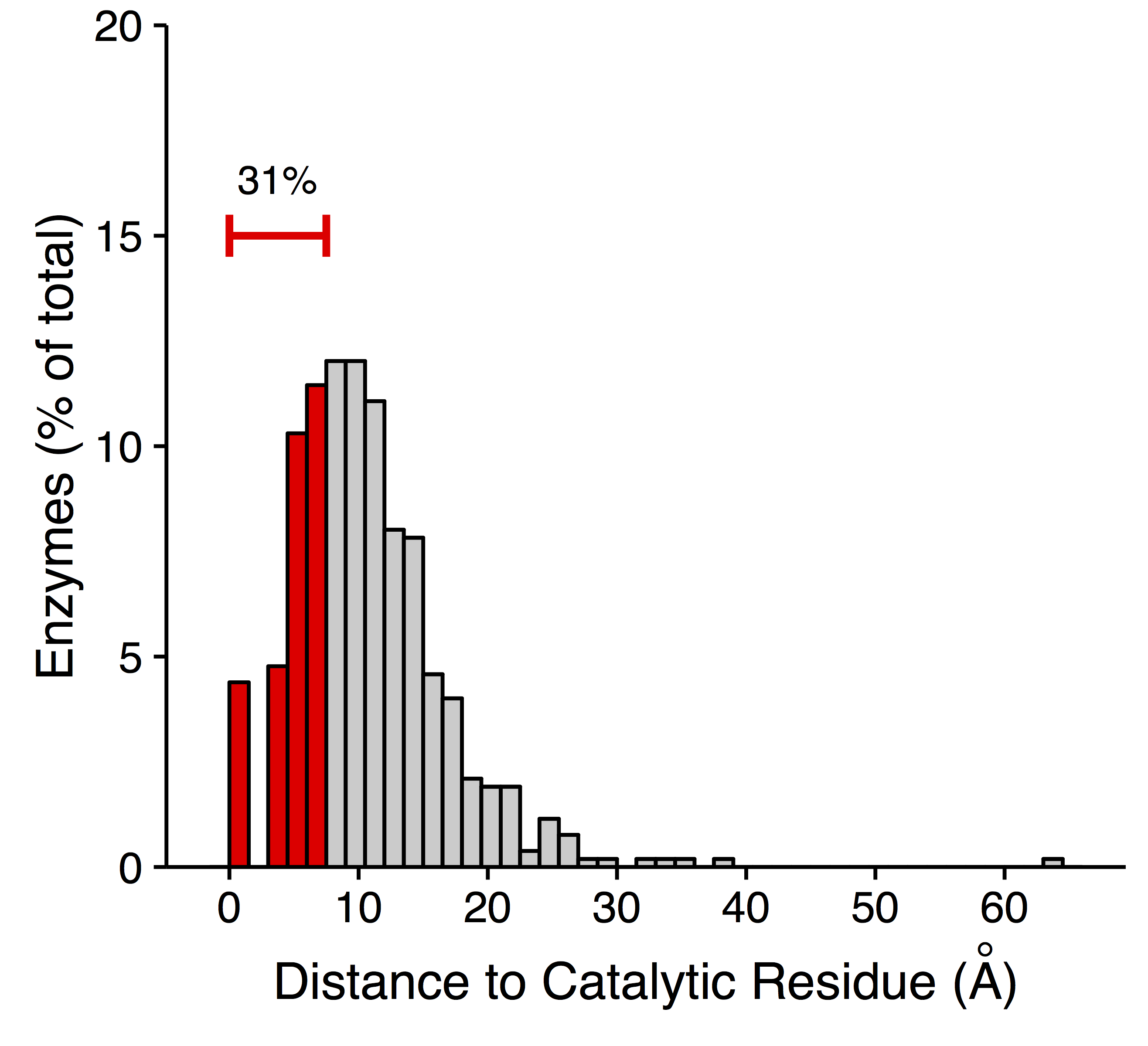

Supplement: S6 Fig — The site with the maximum WCN in a structure is a catalytic residue or a direct contact of a catalytic residue in 31% of enzymes in the dataset. Data underlying this figure are available on Github: https://github.com/benjaminjack/enzyme_distance/tree/master/figure_data. (TIFF) [file pbio.1002452.s009.tiff]

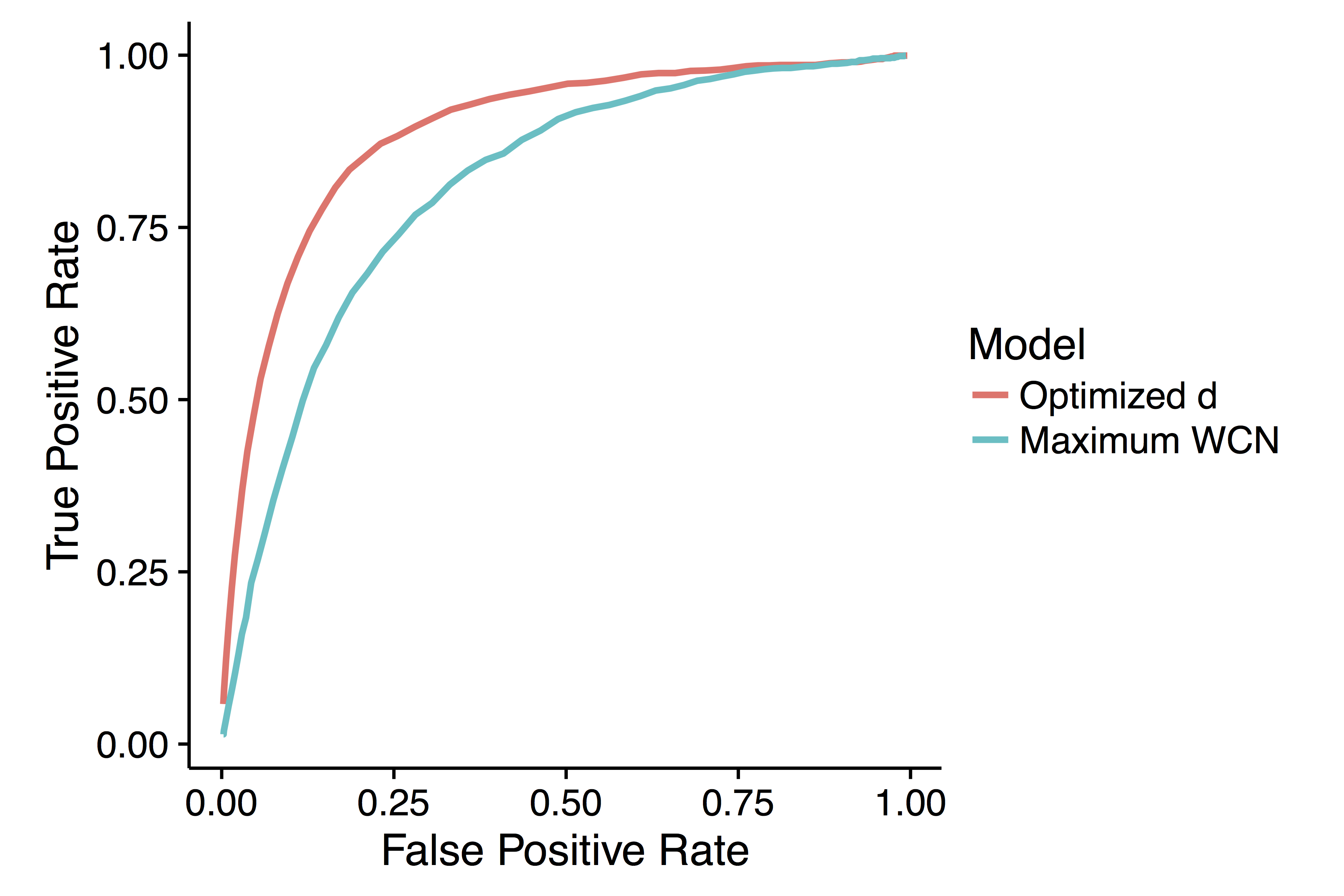

Supplement: S7 Fig — In the optimized d model, we select a residue as a reference residue and then regress rate against the set of distances to that reference residue. We record the R2 and then repeat the process until every residue in the structure has been selected as a reference residue. The predicted catalytic residue is then the reference residue that yields a model with the maximum R2. As a control, we include a maximum WCN model that places the active site at the protein core. The optimized d model (AUC = 0.889) outperforms the maximum WCN model (AUC = 0.809). Data underlying this figure are available on Github: https://github.com/benjaminjack/enzyme_distance/tree/master/figure_data. (TIFF) [file pbio.1002452.s010.tiff]

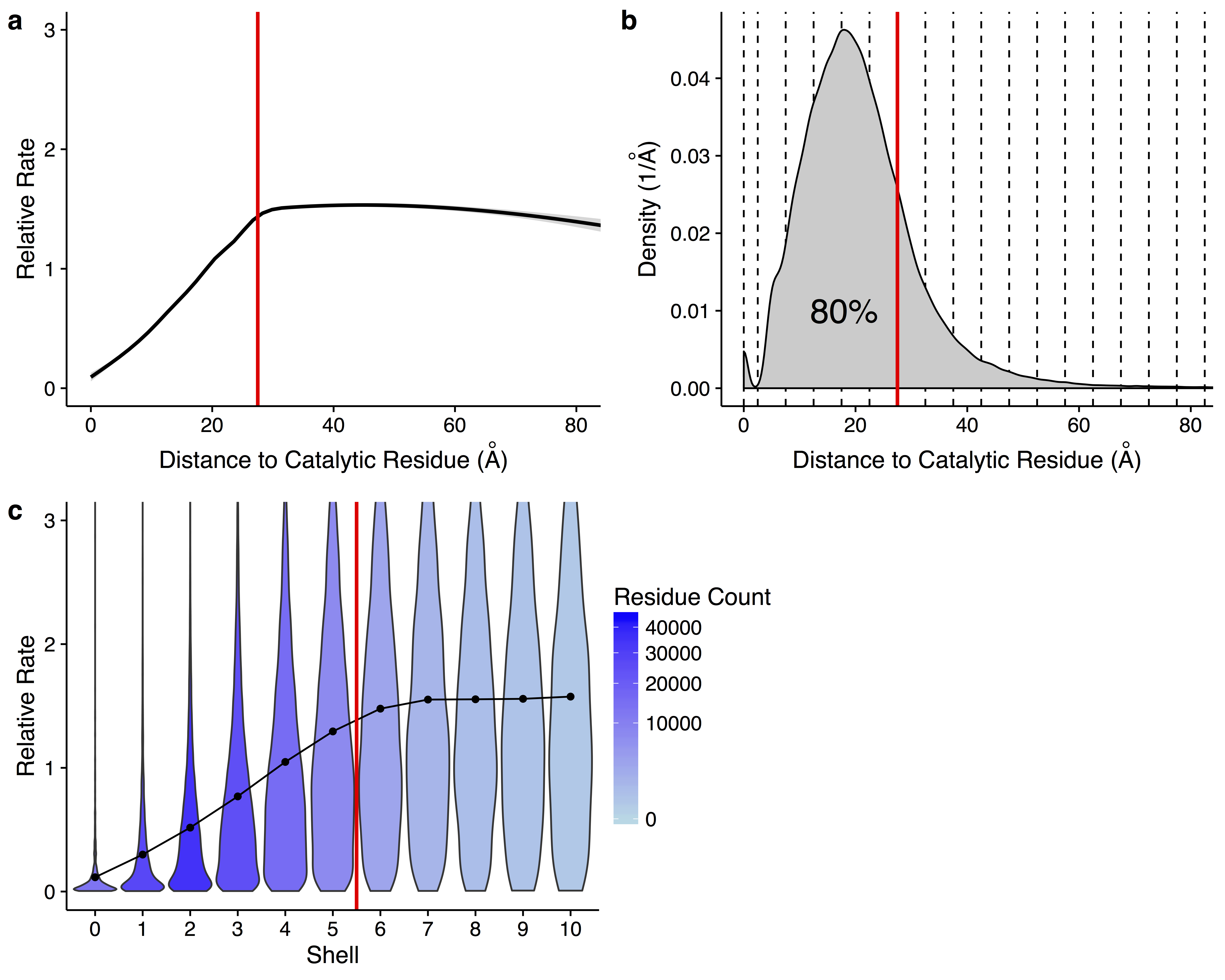

Supplement: S8 Fig — As in Fig 1, but using single subunits with interface residues included. Data underlying this figure are available on Github: https://github.com/benjaminjack/enzyme_distance/tree/master/figure_data. (TIFF) [file pbio.1002452.s011.tiff]

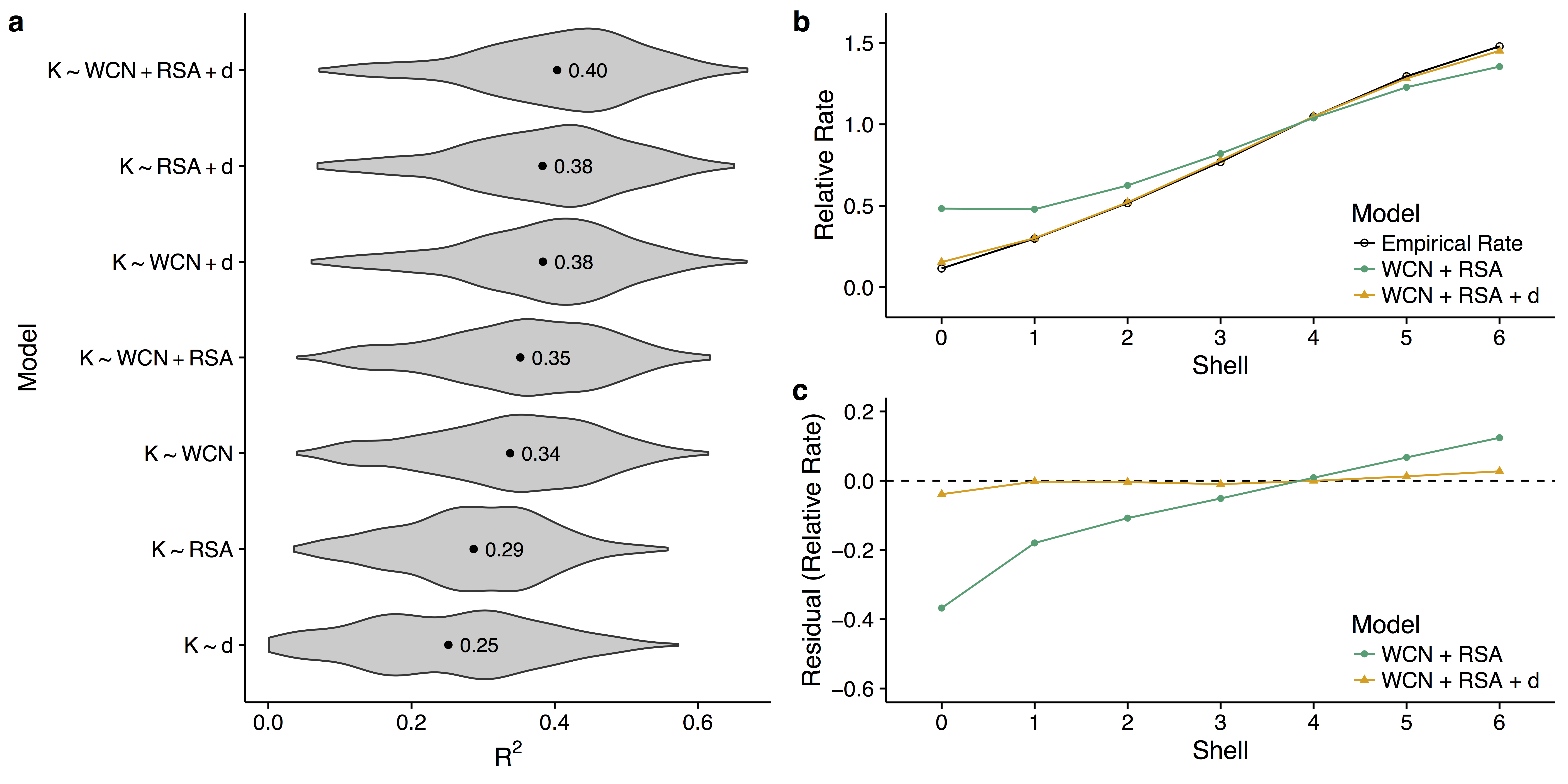

Supplement: S9 Fig — As in Fig 2, but using single subunits with interface residues included. Data underlying this figure are available on Github: https://github.com/benjaminjack/enzyme_distance/tree/master/figure_data. (TIFF) [file pbio.1002452.s012.tiff]

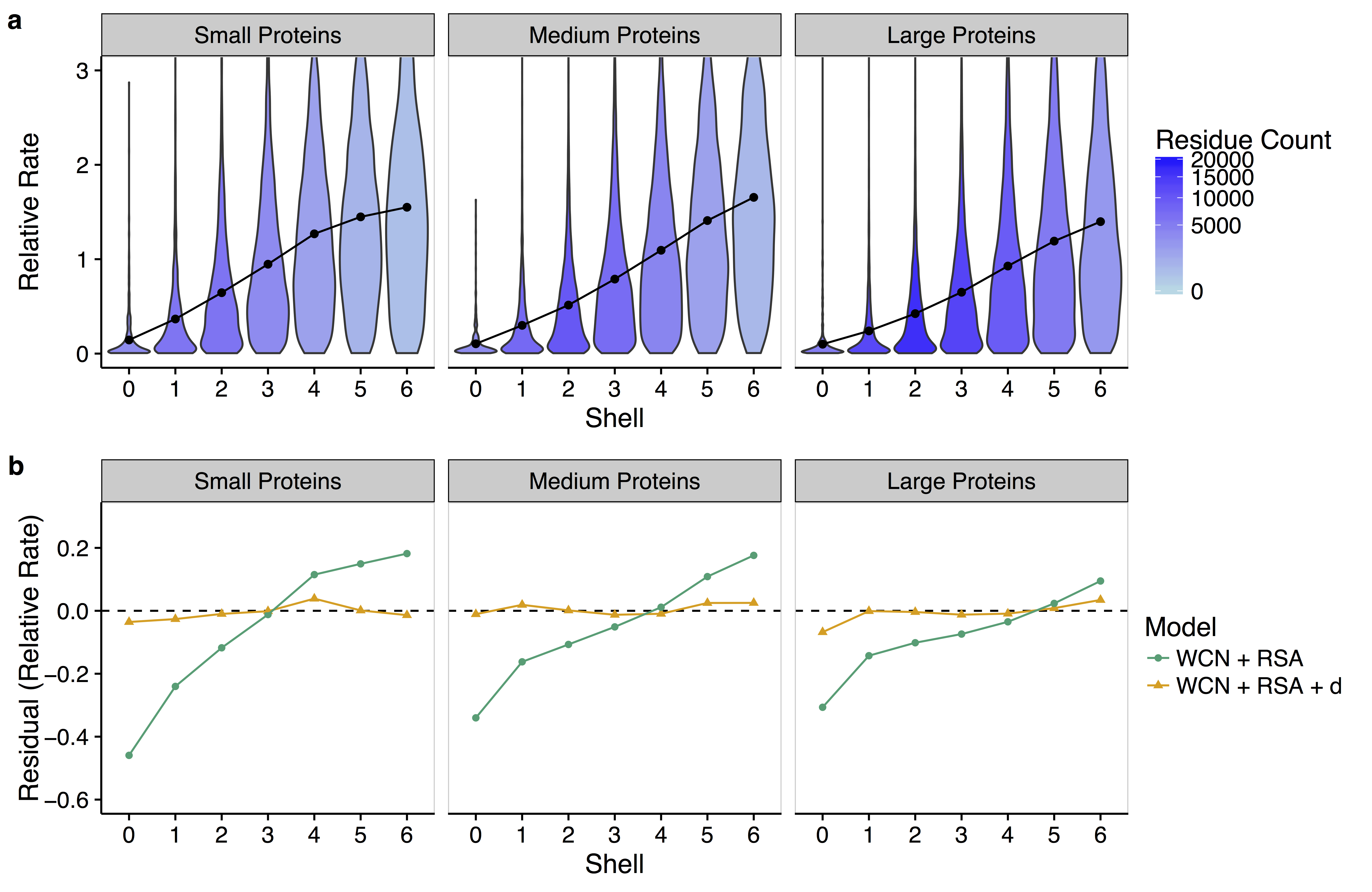

Supplement: S10 Fig — As in Fig 5, but using single subunits with interface residues included. Data underlying this figure are available on Github: https://github.com/benjaminjack/enzyme_distance/tree/master/figure_data. (TIFF) [file pbio.1002452.s013.tiff]

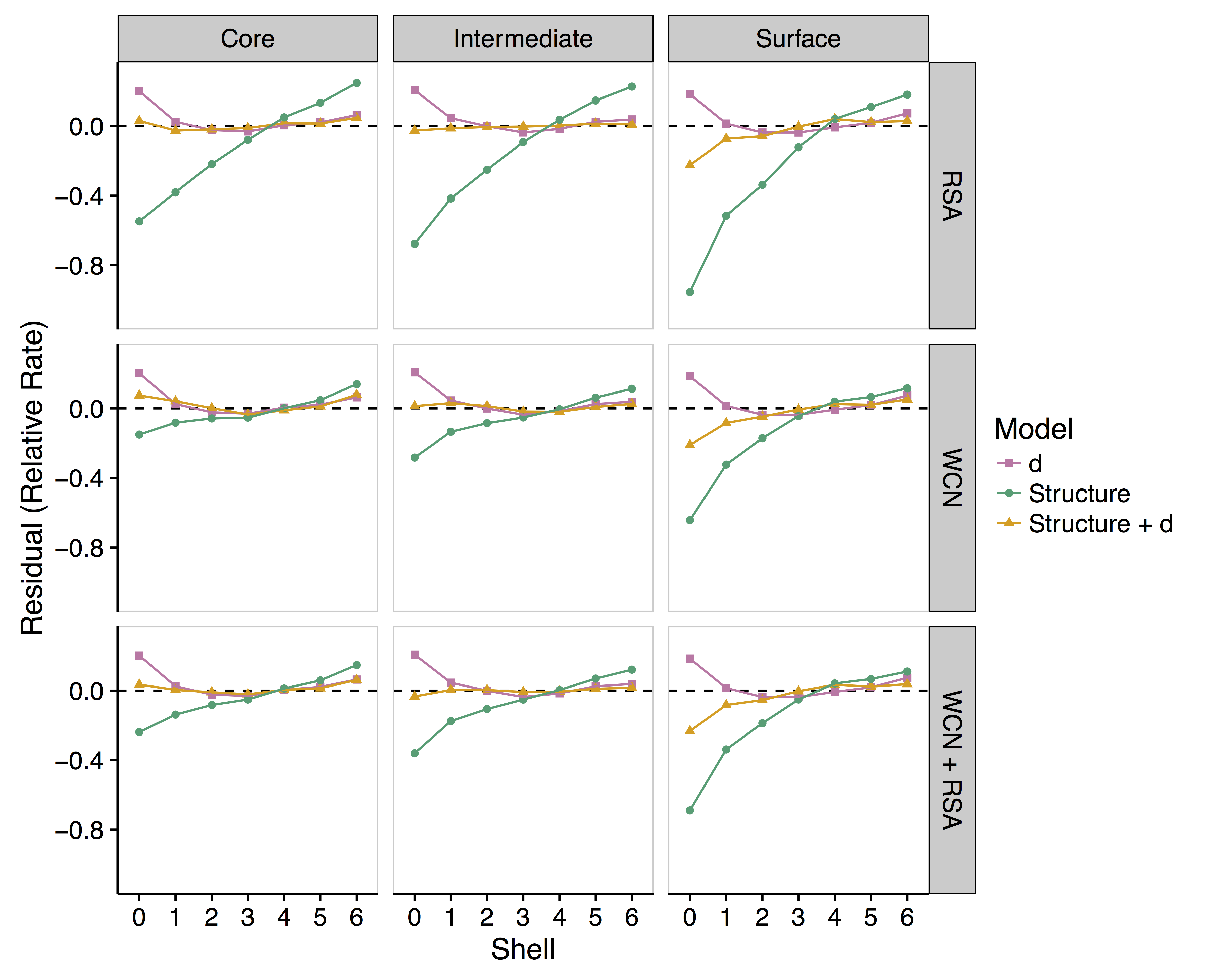

Supplement: S11 Fig — As in S4 Fig, but using single subunits with interface residues included. Data underlying this figure are available on Github: https://github.com/benjaminjack/enzyme_distance/tree/master/figure_data. (TIFF) [file pbio.1002452.s014.tiff]

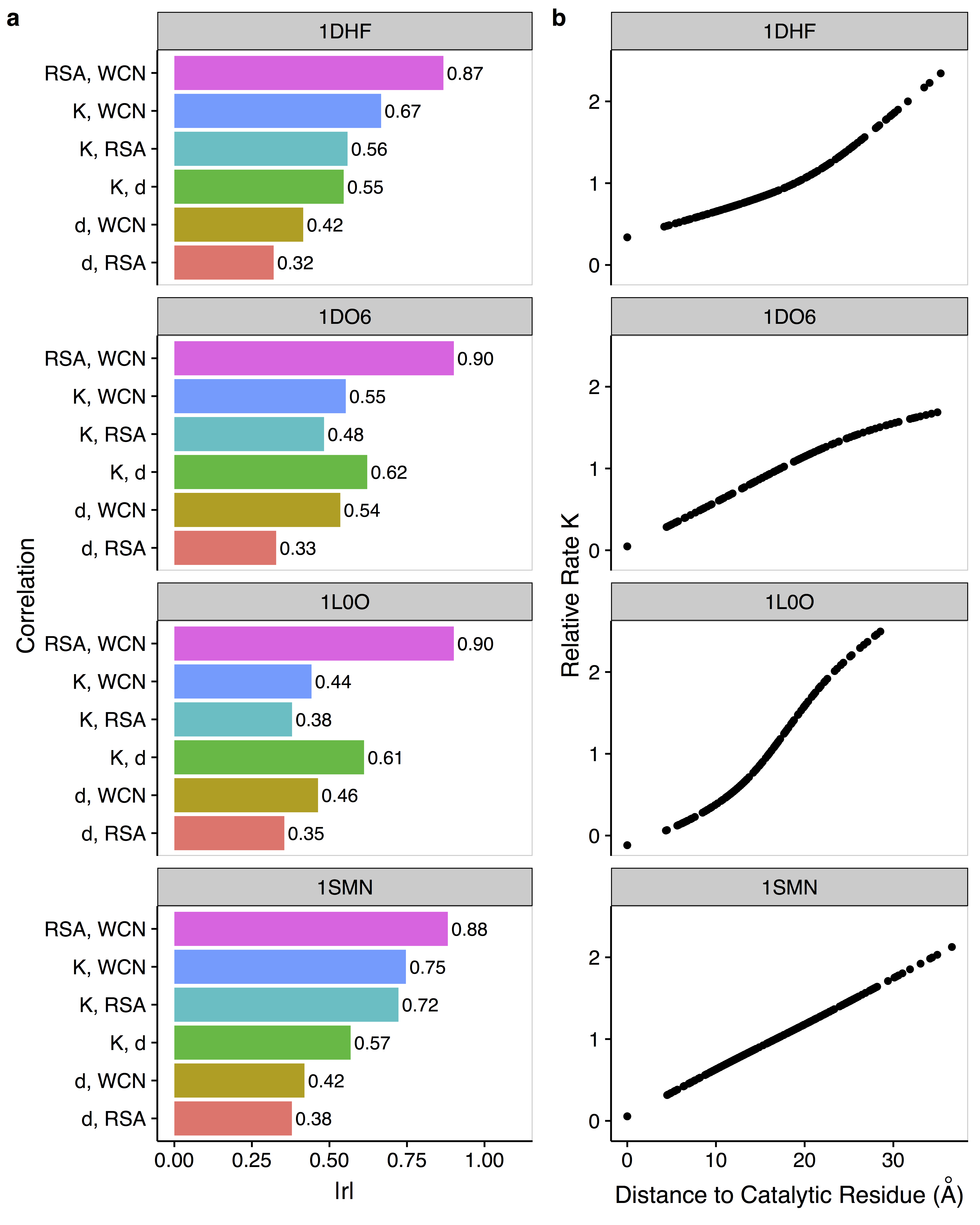

Supplement: S12 Fig — From top to bottom, the PDB IDs of the enzymes structures shown are 1DHF, 1DO6, 1L0O, and 1SMN. As in Fig 3, but using single subunits with interface residues included. Data underlying this figure are available on Github: https://github.com/benjaminjack/enzyme_distance/tree/master/figure_data. (TIFF) [file pbio.1002452.s015.tiff]

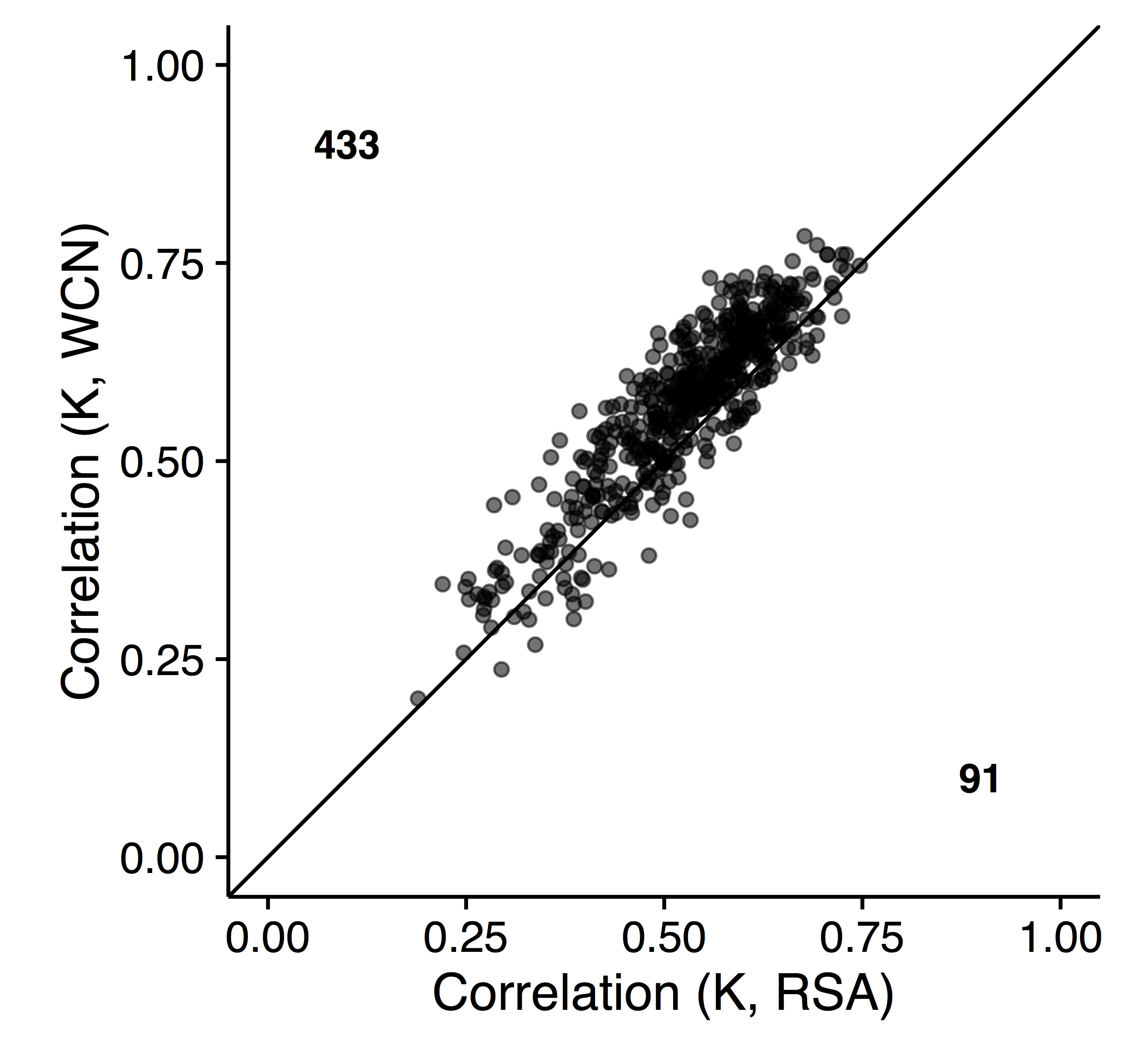

Supplement: S13 Fig — As in S1 Fig, but using single subunits with interface residues included. Data underlying this figure are available on Github: https://github.com/benjaminjack/enzyme_distance/tree/master/figure_data. (TIFF) [file pbio.1002452.s016.tiff]

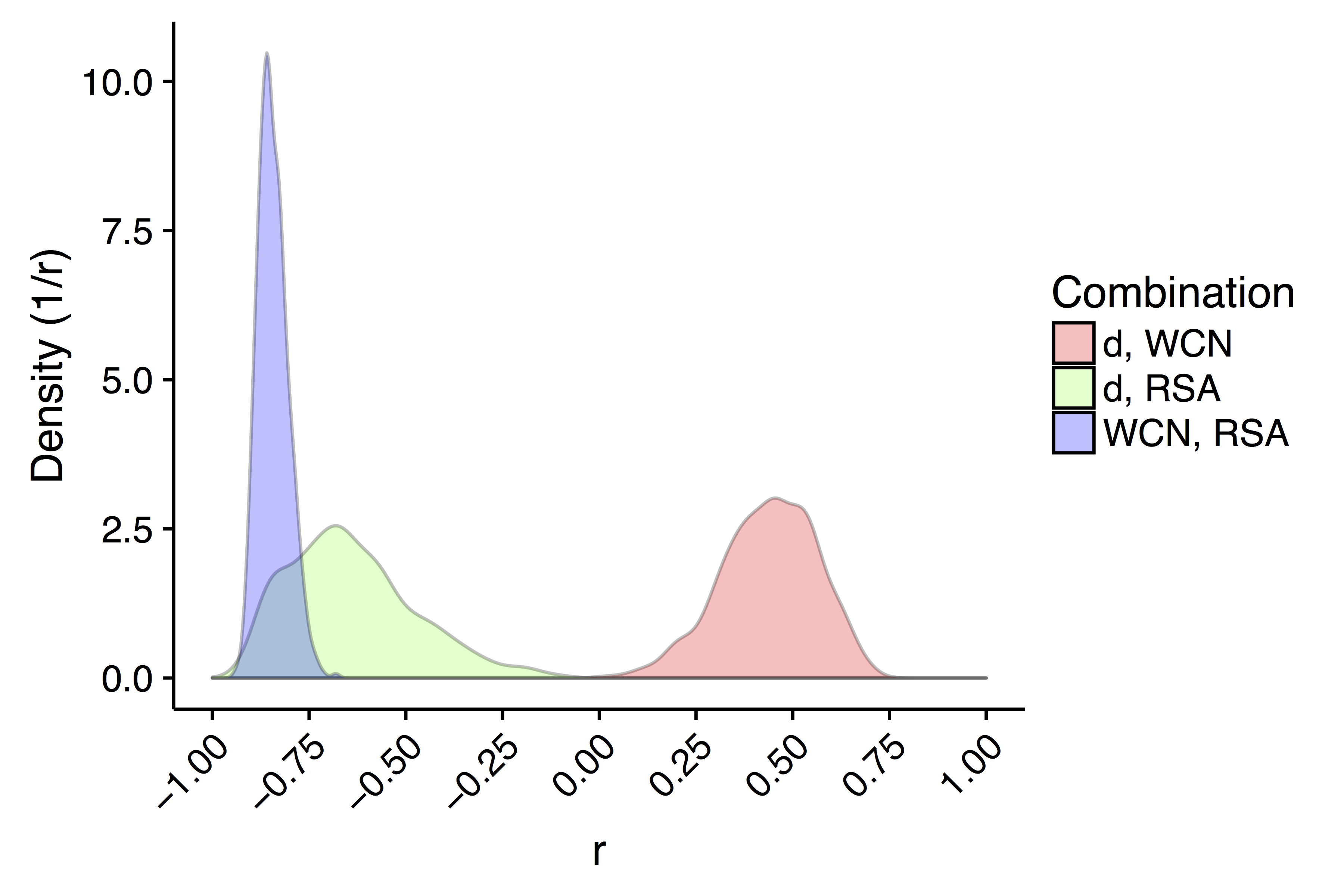

Supplement: S14 Fig — As in S2 Fig, but using single subunits with interface residues included. Data underlying this figure are available on Github: https://github.com/benjaminjack/enzyme_distance/tree/master/figure_data. (TIFF) [file pbio.1002452.s017.tiff]

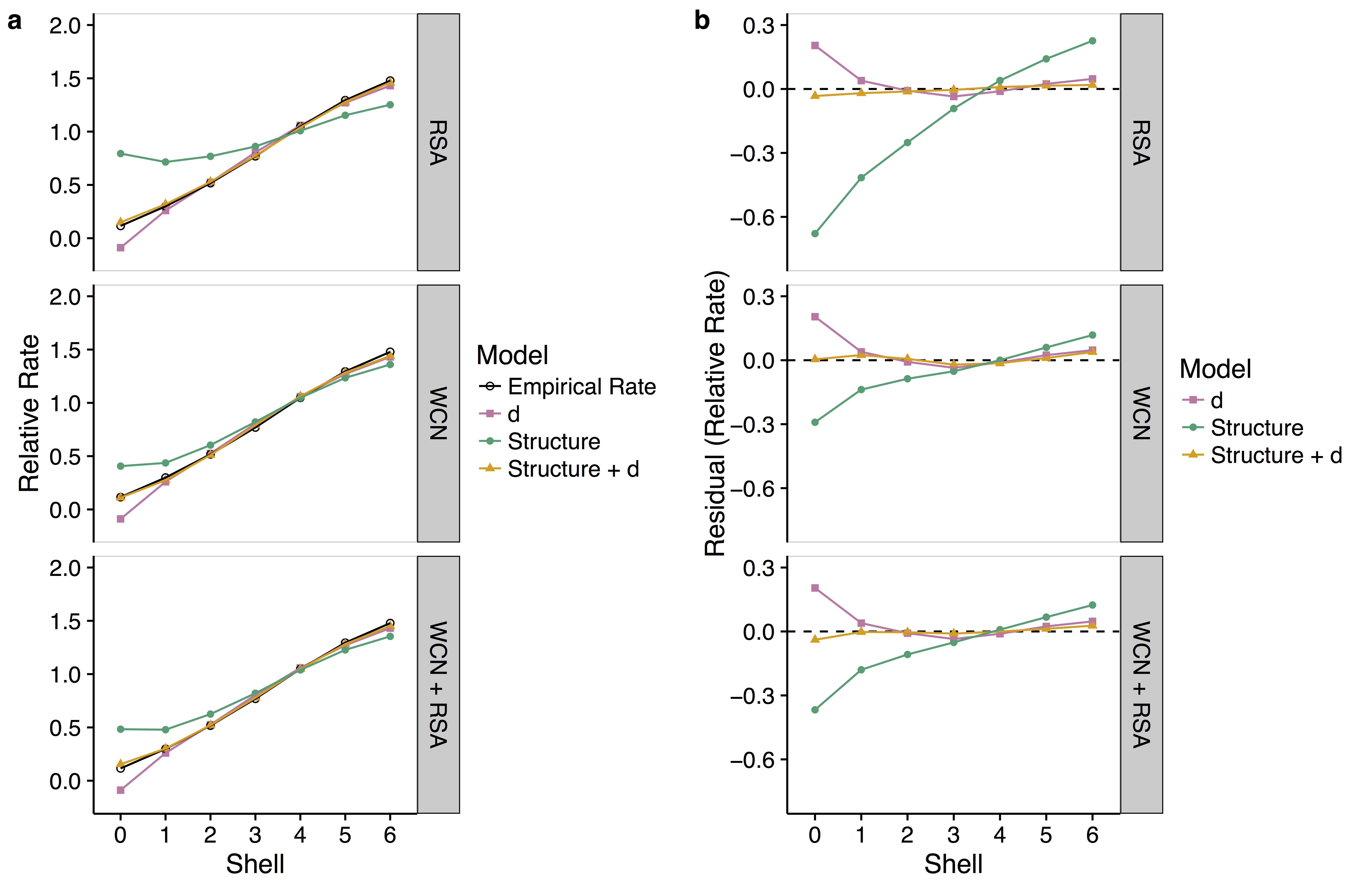

Supplement: S15 Fig — As in S3 Fig, but using single subunits with interface residues included. Data underlying this figure are available on Github: https://github.com/benjaminjack/enzyme_distance/tree/master/figure_data. (TIFF) [file pbio.1002452.s018.tiff]

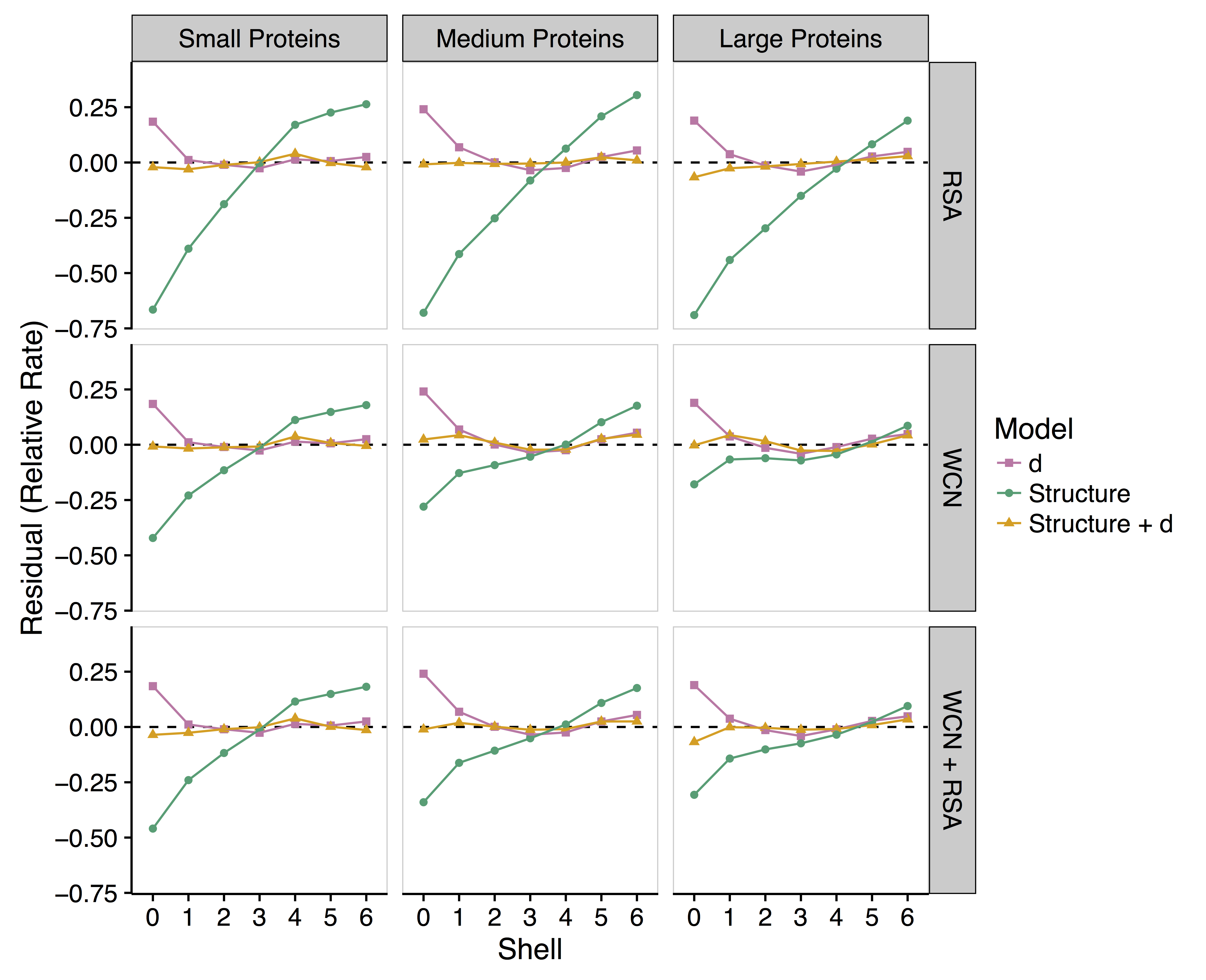

Supplement: S16 Fig — As in S5 Fig, but using single subunits with interface residues included. Data underlying this figure are available on Github: https://github.com/benjaminjack/enzyme_distance/tree/master/figure_data. (TIFF) [file pbio.1002452.s019.tiff]

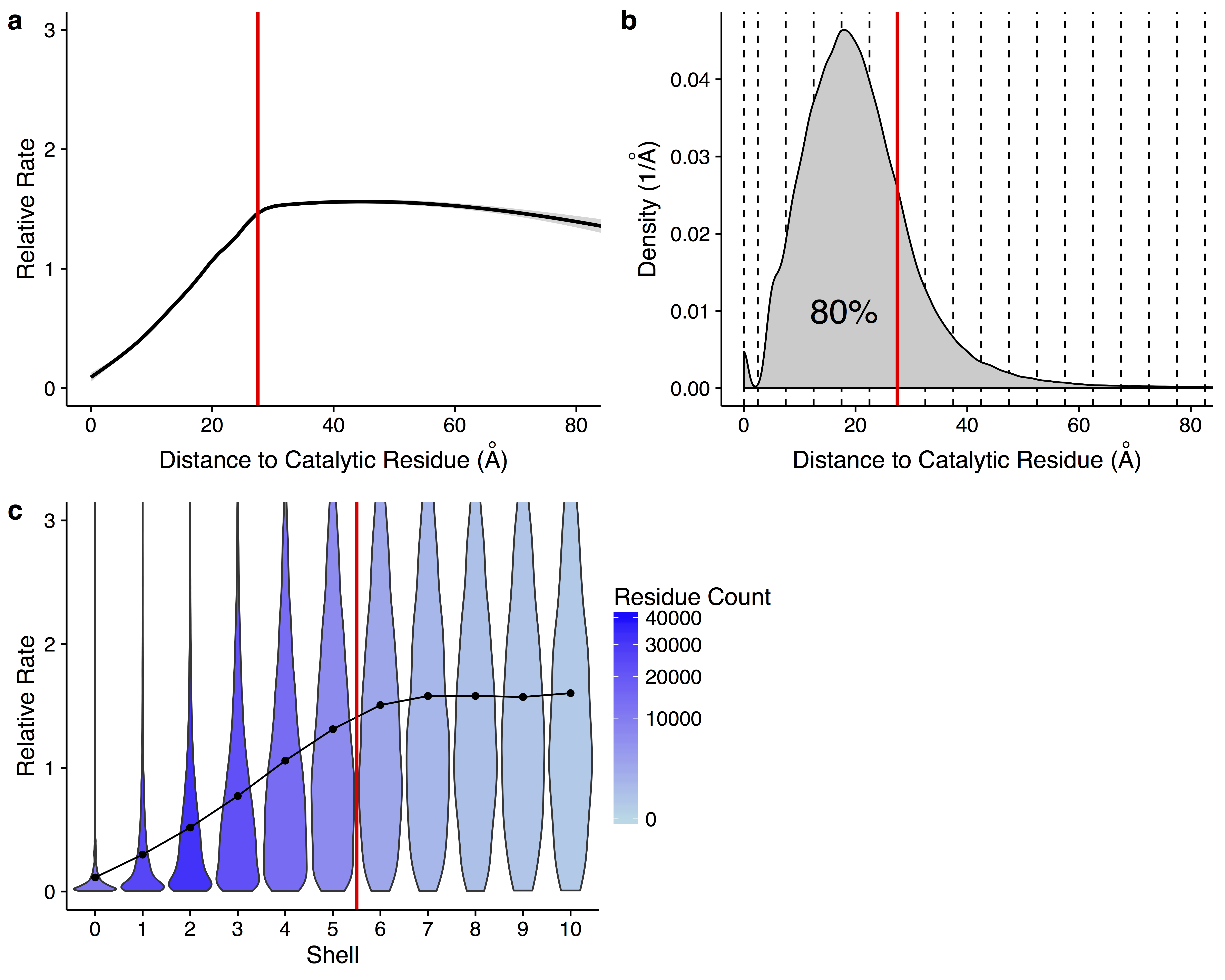

Supplement: S17 Fig — As in Fig 1, but using single subunits with interface residues removed. Data underlying this figure are available on Github: https://github.com/benjaminjack/enzyme_distance/tree/master/figure_data. (TIFF) [file pbio.1002452.s020.tiff]

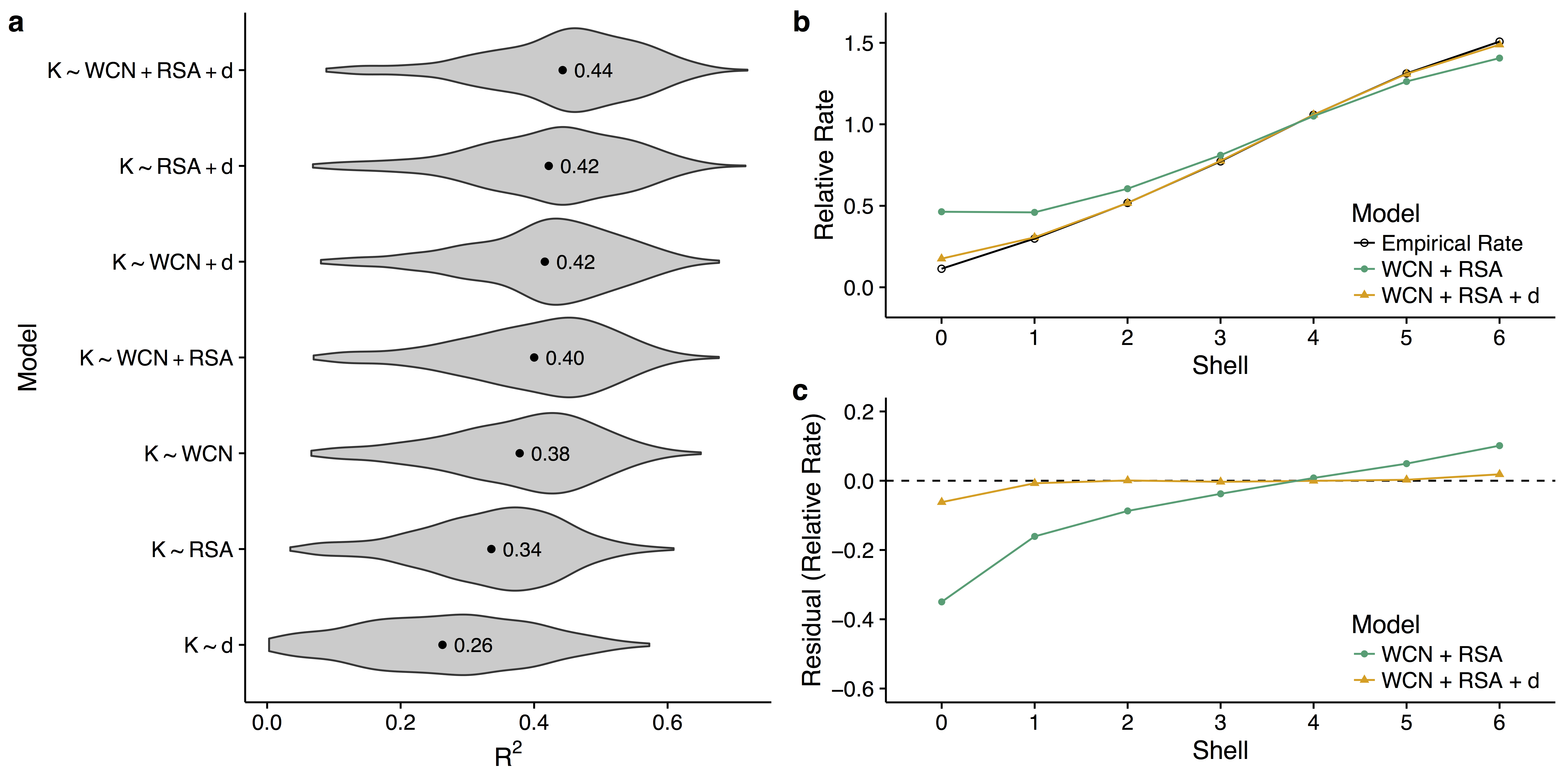

Supplement: S18 Fig — As in Fig 2, but using single subunits with interface residues removed. Data underlying this figure are available on Github: https://github.com/benjaminjack/enzyme_distance/tree/master/figure_data. (TIFF) [file pbio.1002452.s021.tiff]

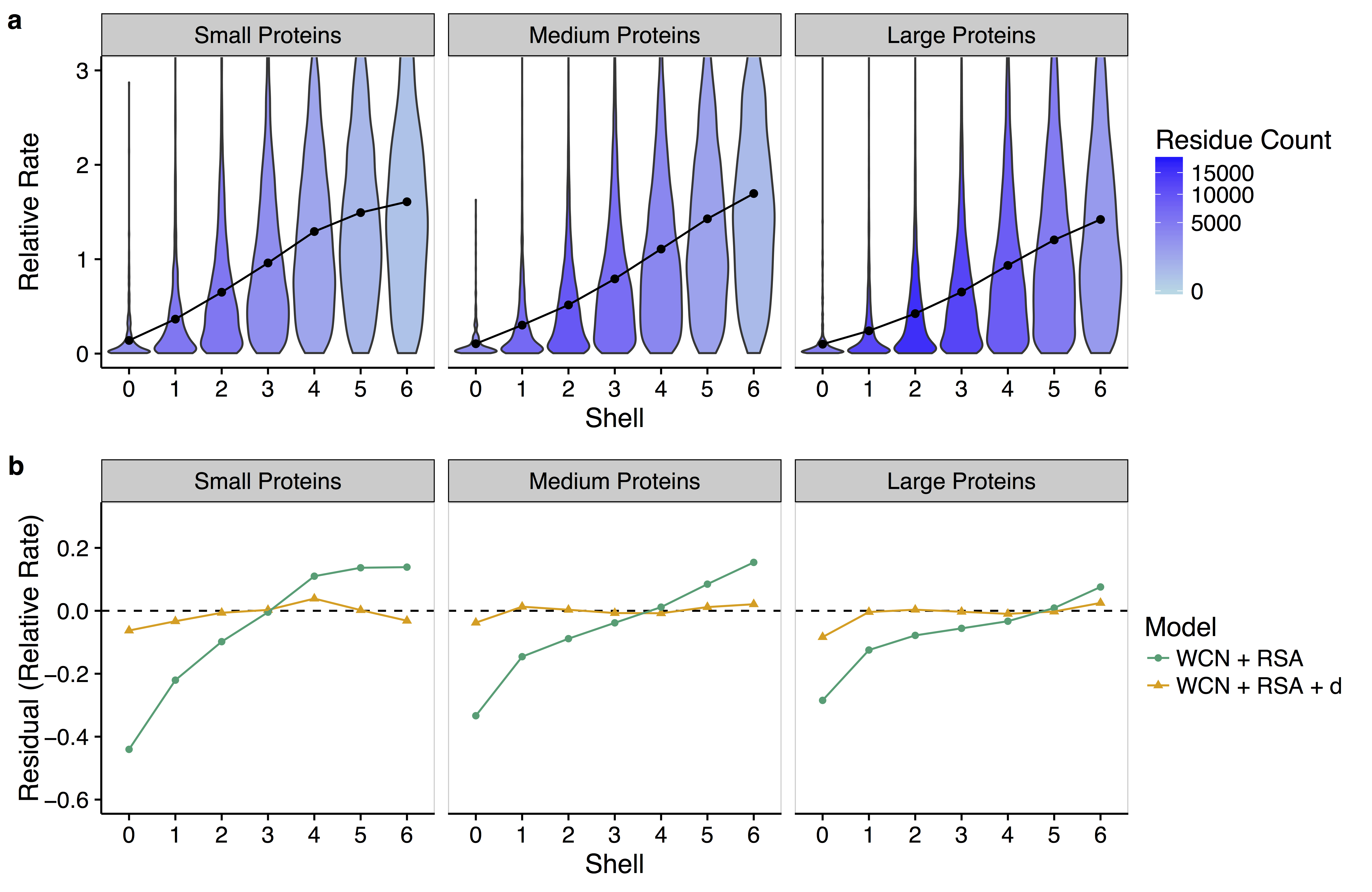

Supplement: S19 Fig — As in Fig 5, but using single subunits with interface residues removed. Data underlying this figure are available on Github: https://github.com/benjaminjack/enzyme_distance/tree/master/figure_data. (TIFF) [file pbio.1002452.s022.tiff]

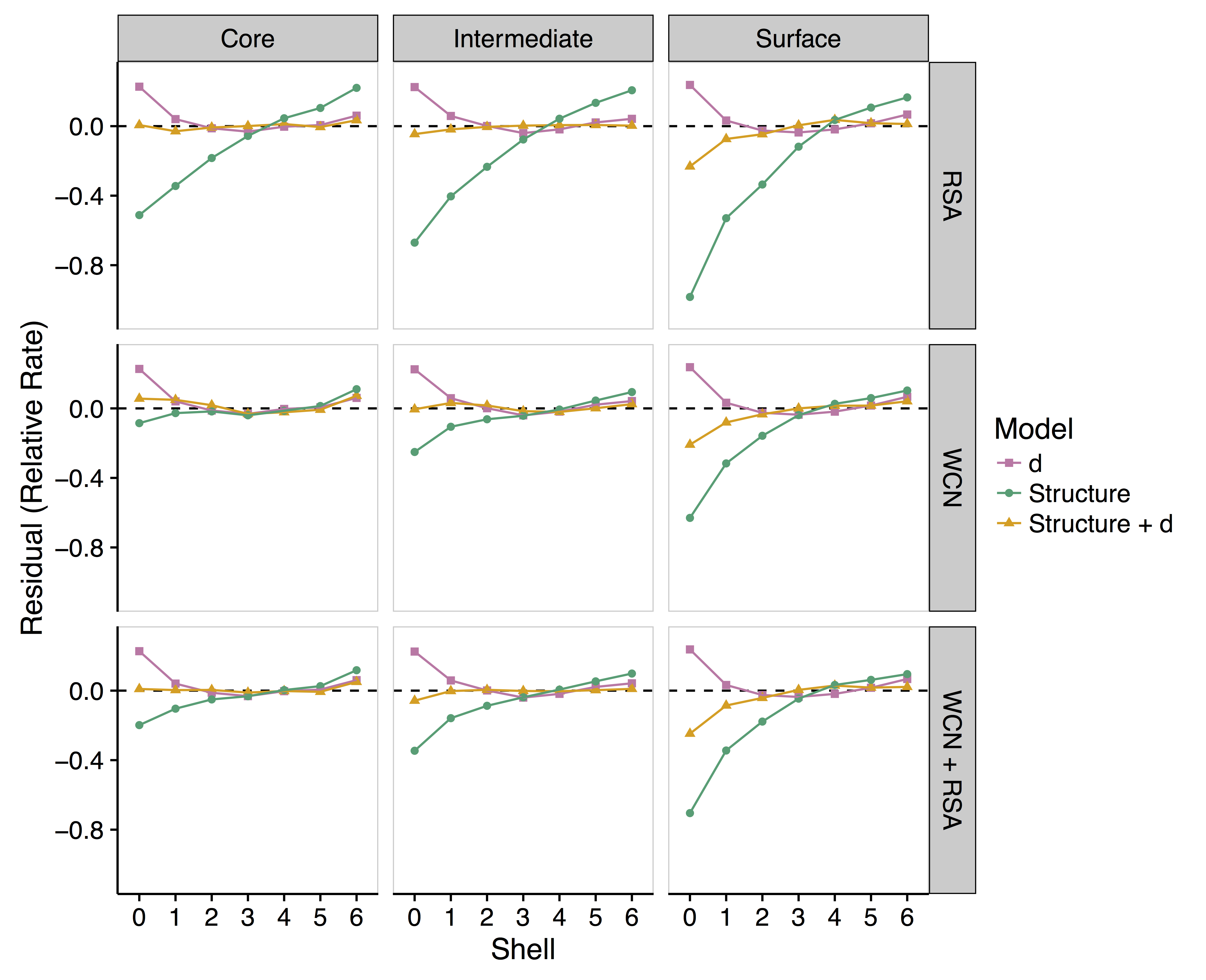

Supplement: S20 Fig — As in Fig 4, but using single subunits with interface residues removed. Data underlying this figure are available on Github: https://github.com/benjaminjack/enzyme_distance/tree/master/figure_data. (TIFF) [file pbio.1002452.s023.tiff]

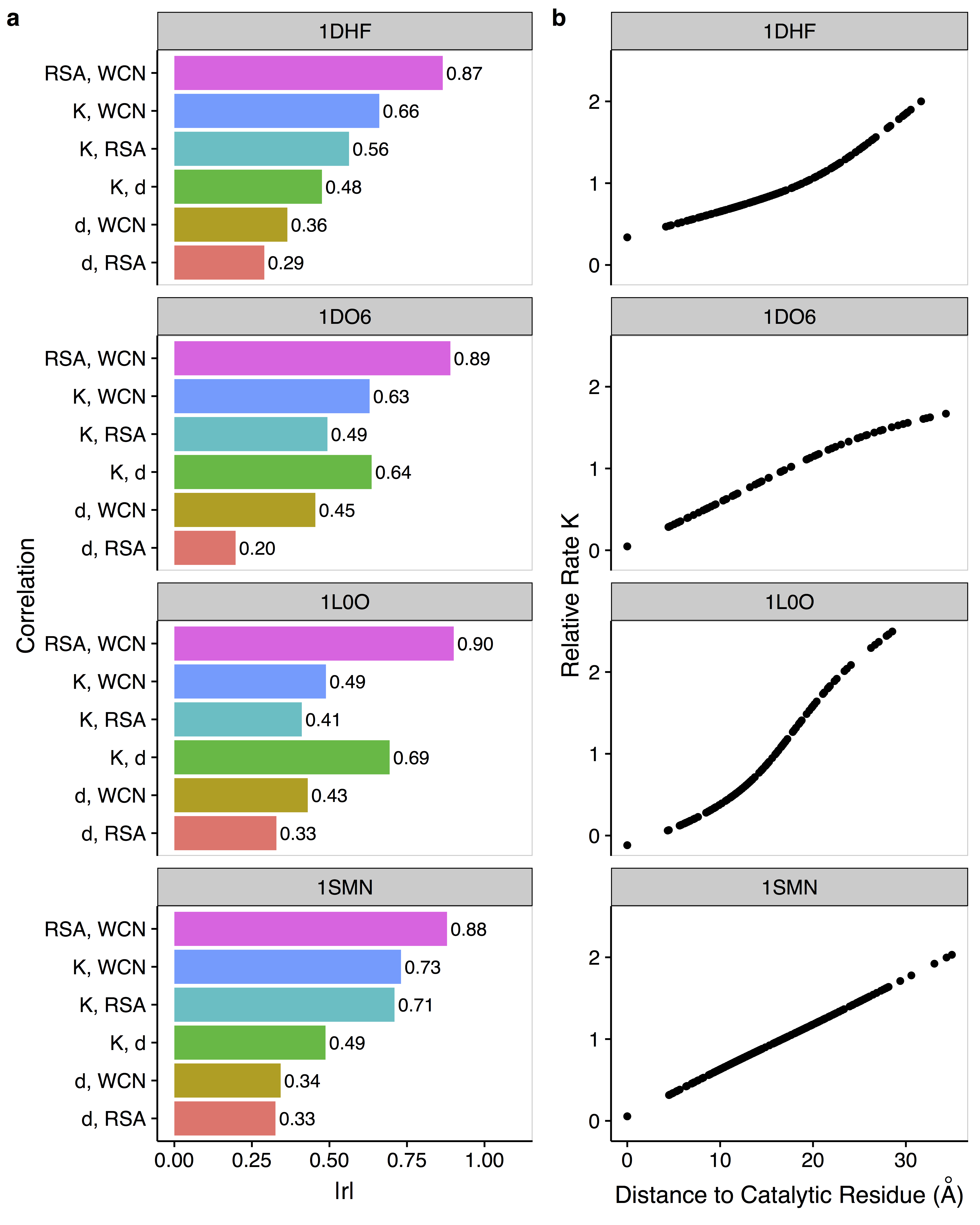

Supplement: S21 Fig — From top to bottom, the PDB IDs of the enzyme structures shown are 1DHF, 1DO6, 1L0O, and 1SMN. As in Fig 3, but using single subunits with interface residues removed. Data underlying this figure are available on Github: https://github.com/benjaminjack/enzyme_distance/tree/master/figure_data. (TIFF) [file pbio.1002452.s024.tiff]

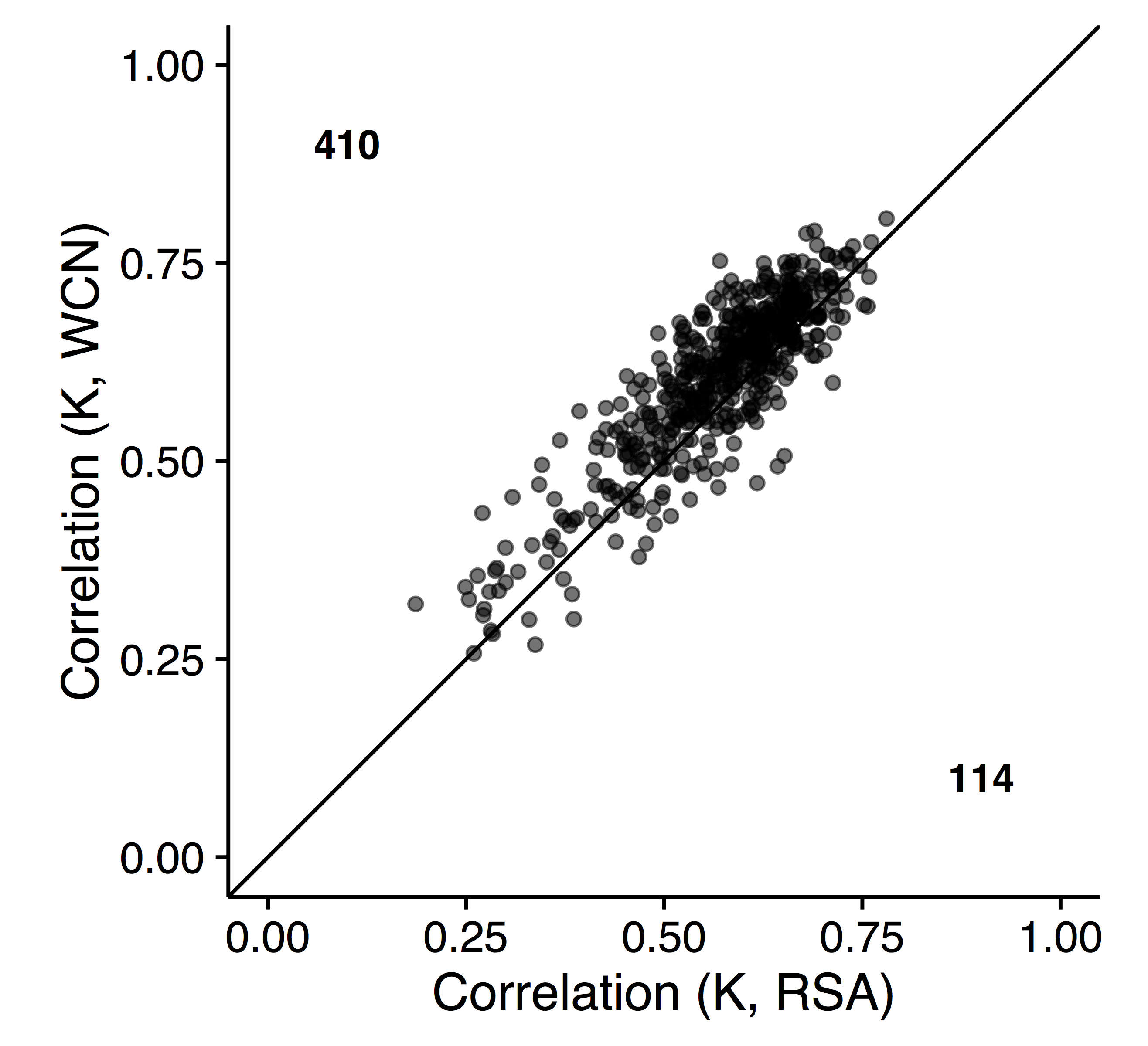

Supplement: S22 Fig — As in S1 Fig, but using single subunits with interface residues removed. Data underlying this figure are available on Github: https://github.com/benjaminjack/enzyme_distance/tree/master/figure_data. (TIFF) [file pbio.1002452.s025.tiff]

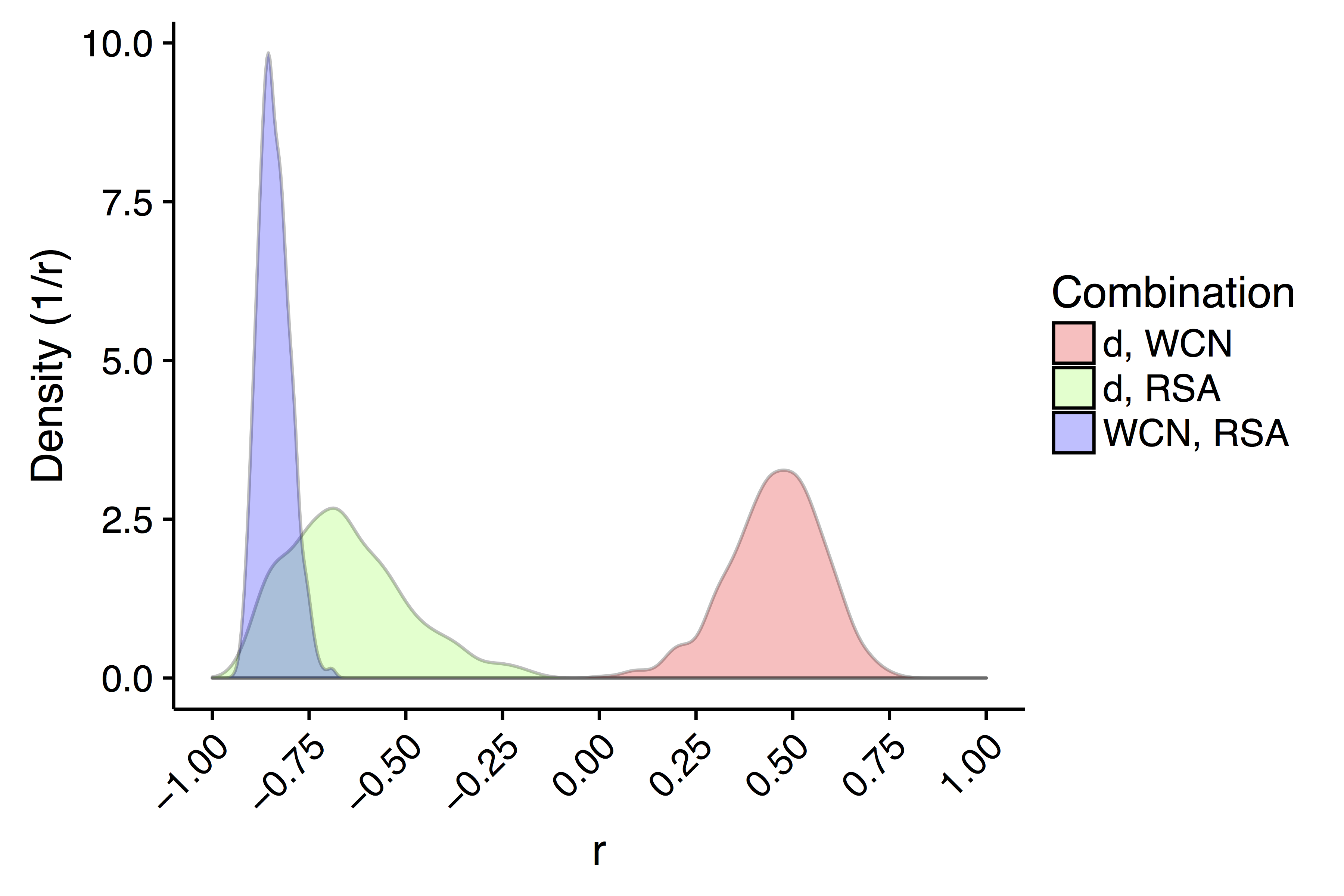

Supplement: S23 Fig — As in S2 Fig, but using single subunits with interface residues removed. Data underlying this figure are available on Github: https://github.com/benjaminjack/enzyme_distance/tree/master/figure_data. (TIFF) [file pbio.1002452.s026.tiff]

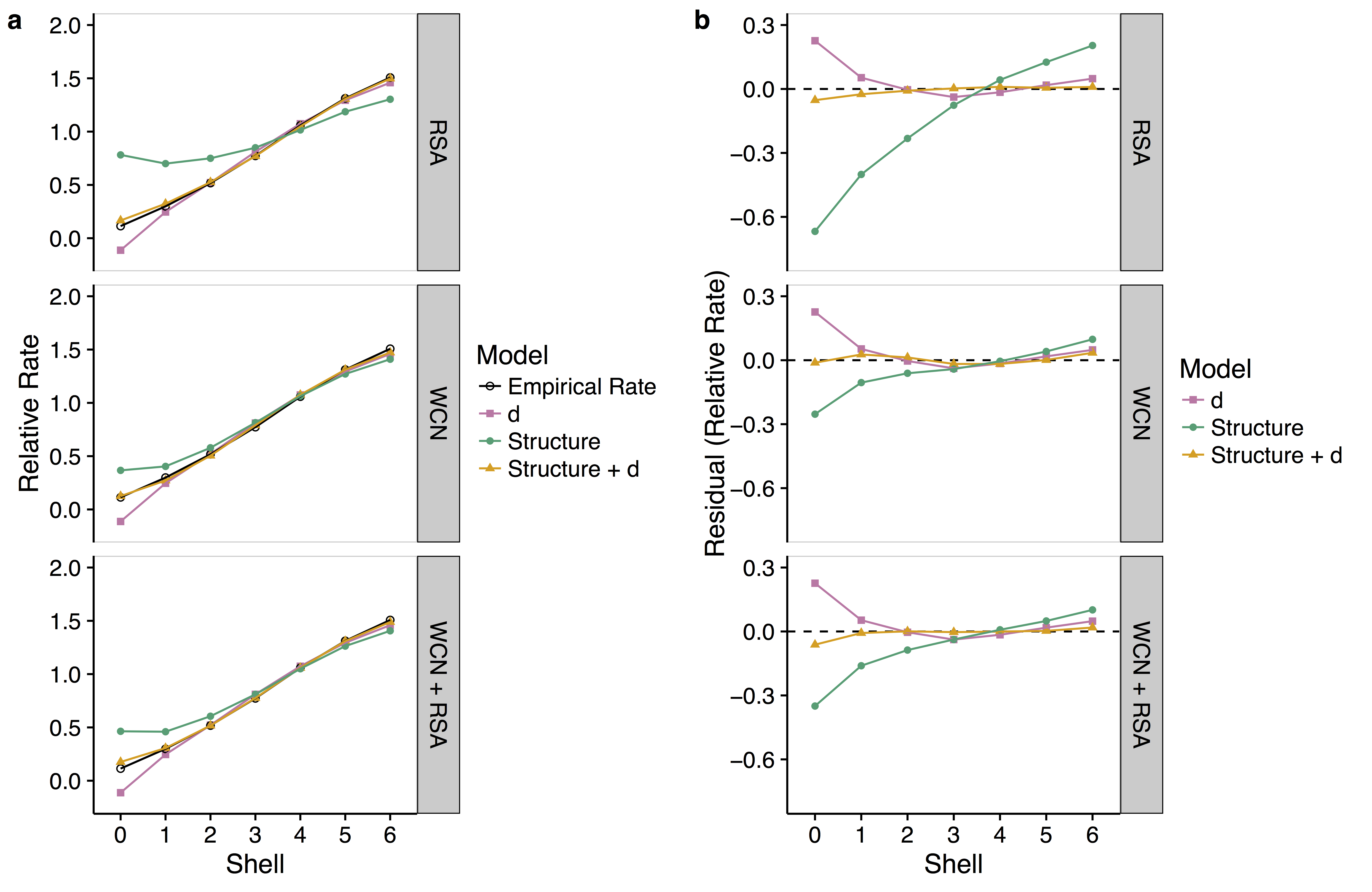

Supplement: S24 Fig — As in S3 Fig, but using single subunits with interface residues removed. Data underlying this figure are available on Github: https://github.com/benjaminjack/enzyme_distance/tree/master/figure_data. (TIFF) [file pbio.1002452.s027.tiff]

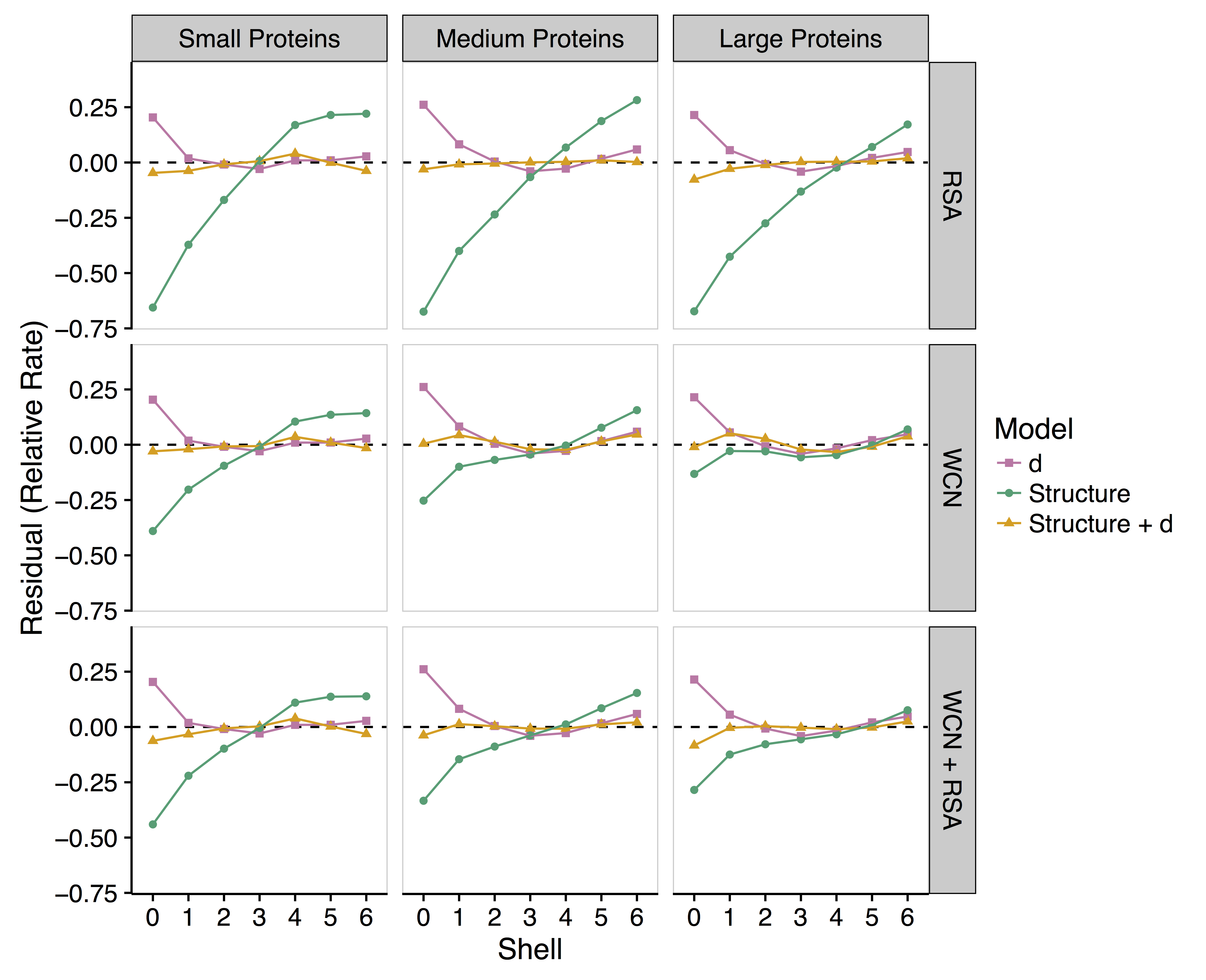

Supplement: S25 Fig — As in S5 Fig, but using single subunits with interface residues removed. Data underlying this figure are available on Github: https://github.com/benjaminjack/enzyme_distance/tree/master/figure_data. (TIFF) [file pbio.1002452.s028.tiff]

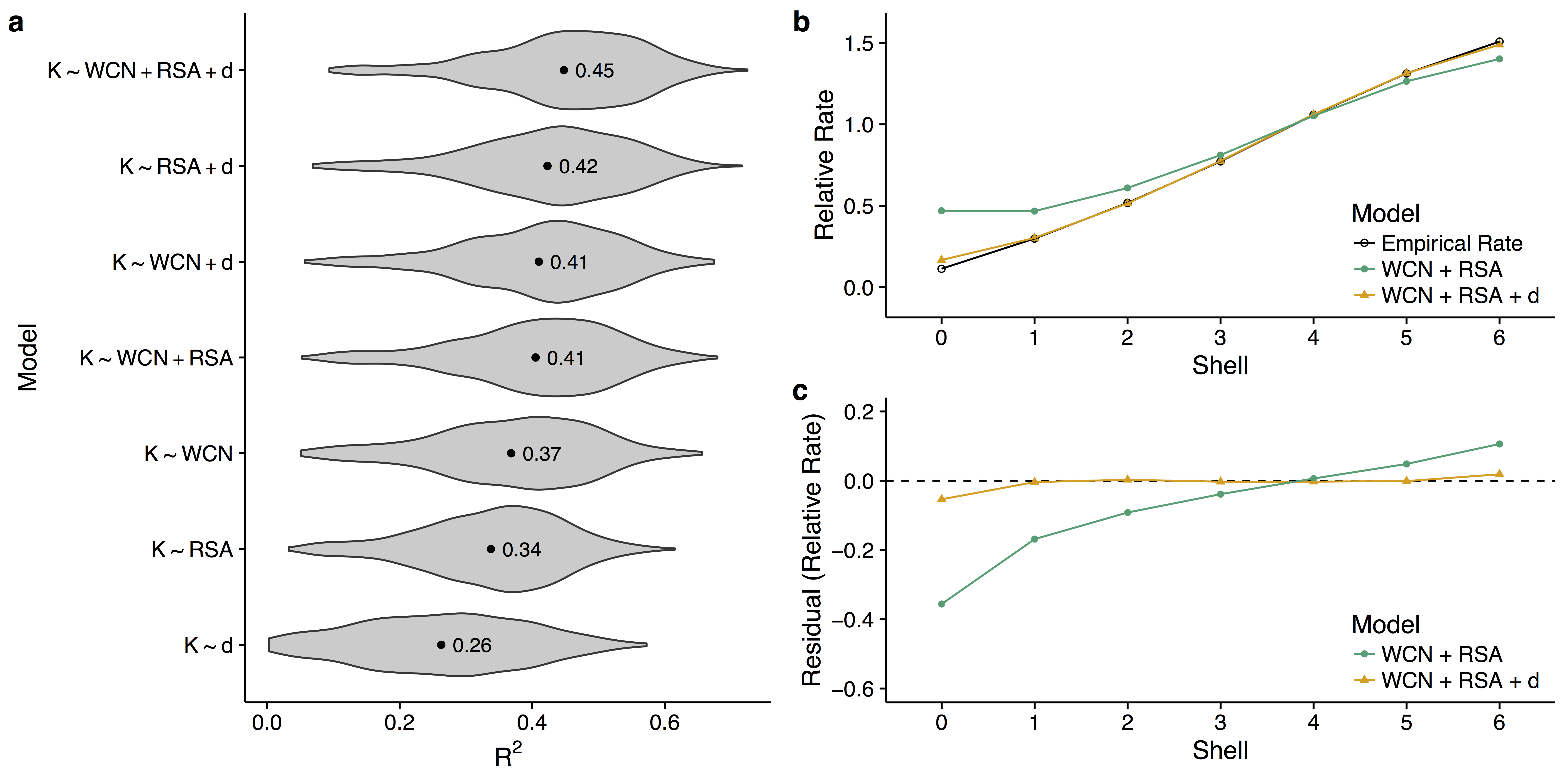

Supplement: S27 Fig — As in Fig 2, but using biological assemblies with interface residues removed. Data underlying this figure are available on Github: https://github.com/benjaminjack/enzyme_distance/tree/master/figure_data. (TIFF) [file pbio.1002452.s030.tiff]

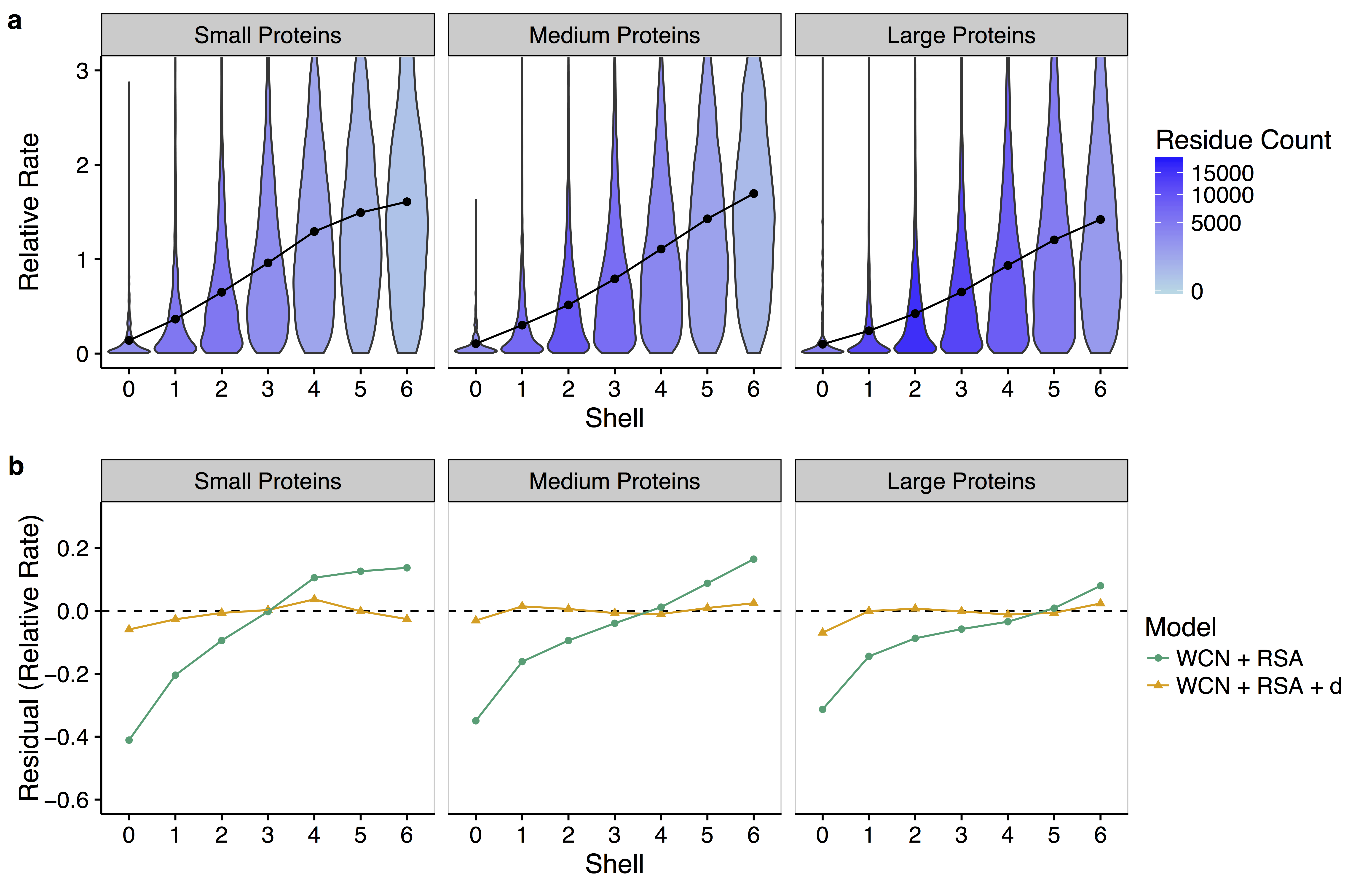

Supplement: S28 Fig — As in Fig 5, but using biological assemblies with interface residues removed. Data underlying this figure are available on Github: https://github.com/benjaminjack/enzyme_distance/tree/master/figure_data. (TIFF) [file pbio.1002452.s031.tiff]

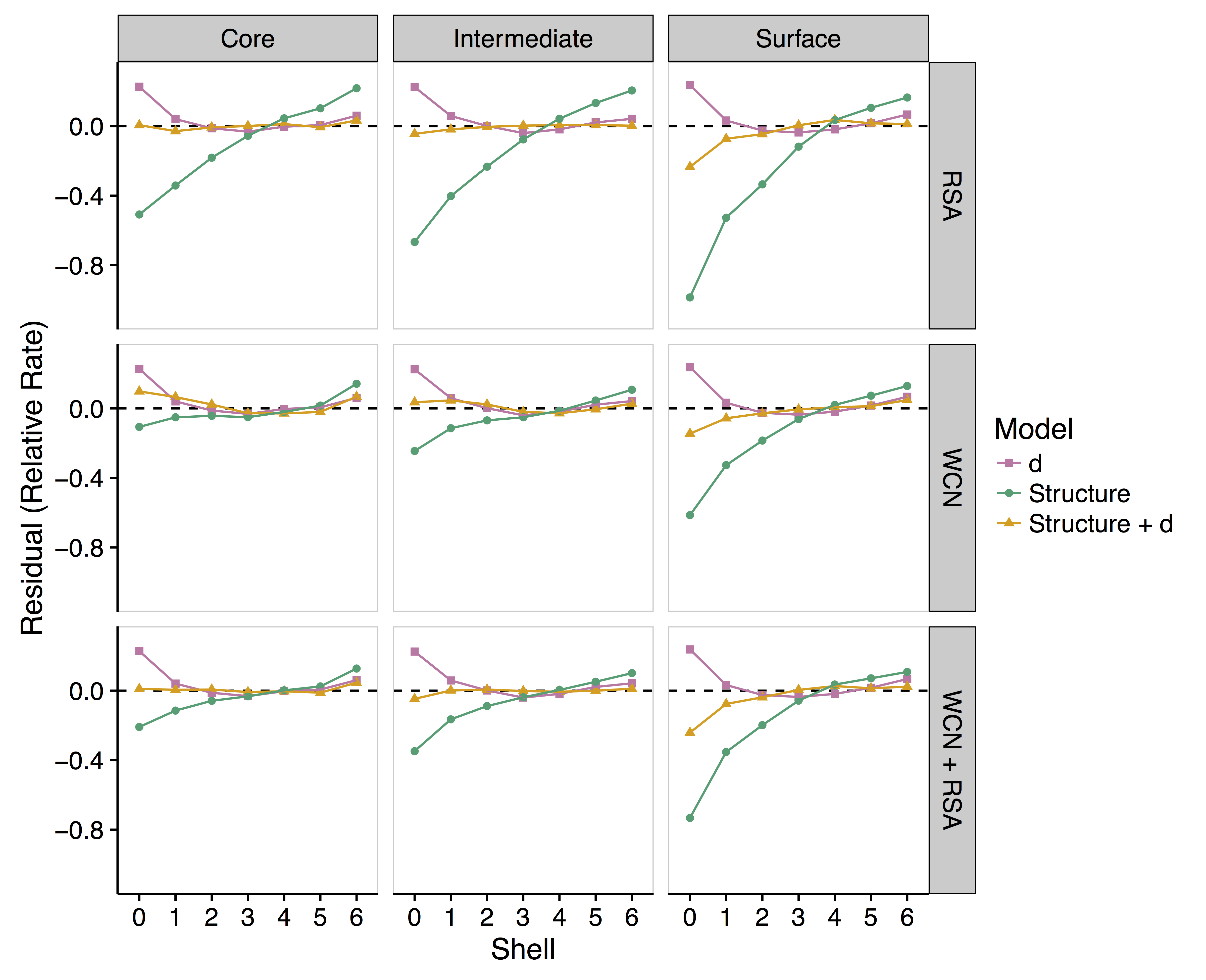

Supplement: S29 Fig — As in Fig 4, but using biological assemblies with interface residues removed. Data underlying this figure are available on Github: https://github.com/benjaminjack/enzyme_distance/tree/master/figure_data. (TIFF) [file pbio.1002452.s032.tiff]

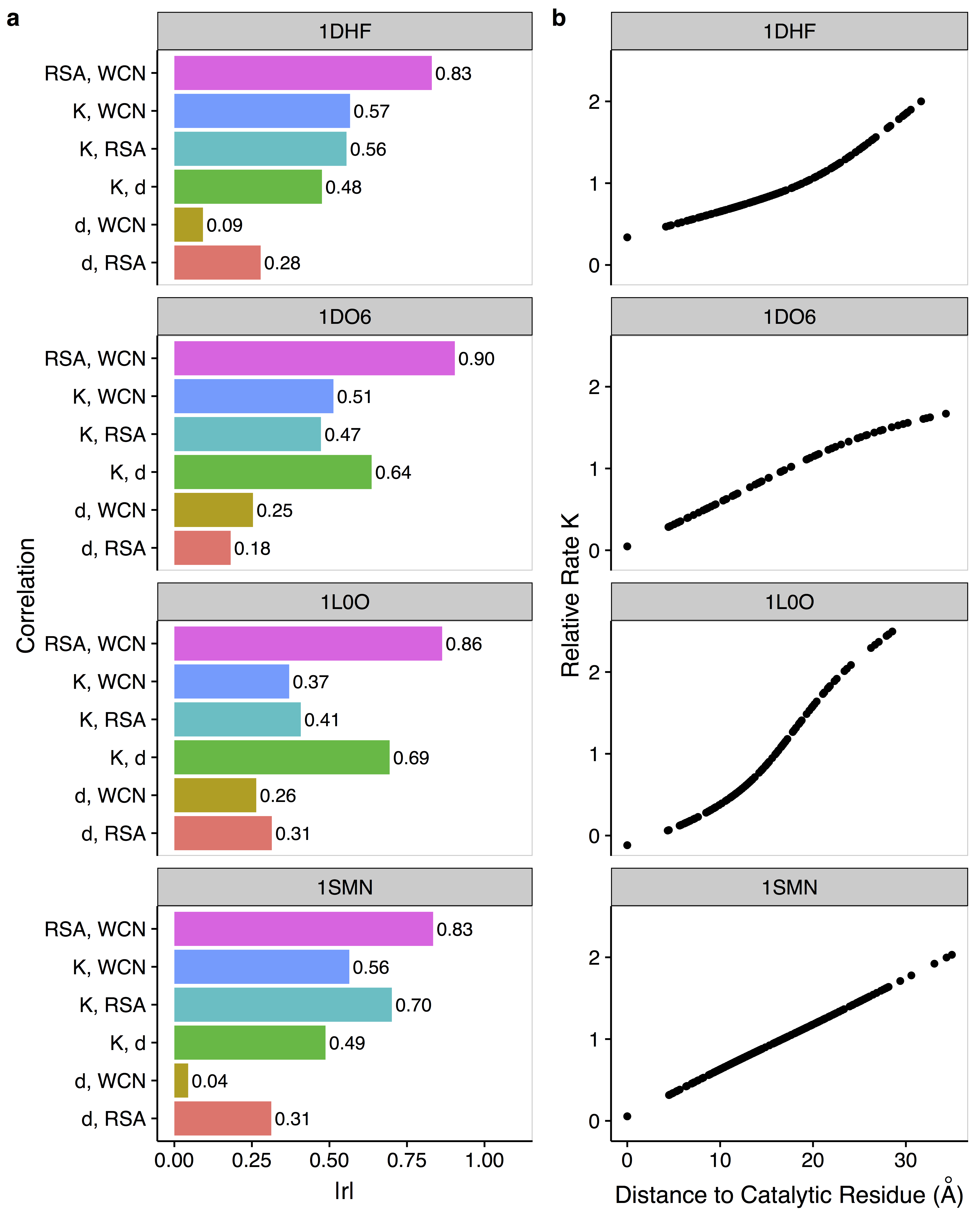

Supplement: S30 Fig — From top to bottom, the PDB IDs of the enzyme structures shown are 1DHF, 1DO6, 1L0O, and 1SMN. As in Fig 3, but using biological assemblies with interface residues removed. Data underlying this figure are available on Github: https://github.com/benjaminjack/enzyme_distance/tree/master/figure_data. (TIFF) [file pbio.1002452.s033.tiff]

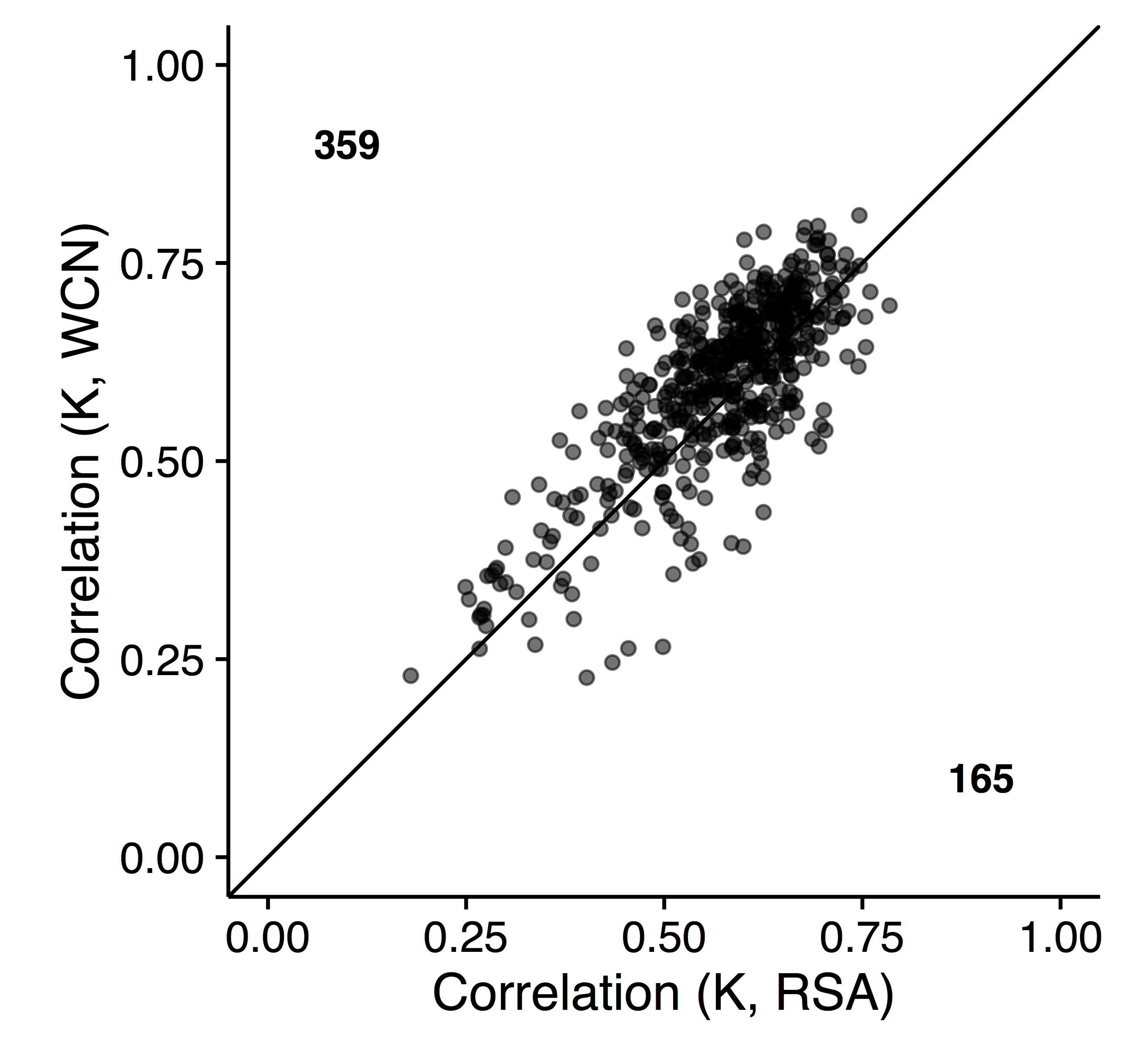

Supplement: S31 Fig — As in S1 Fig, but using biological assemblies with interface residues removed. Data underlying this figure are available on Github: https://github.com/benjaminjack/enzyme_distance/tree/master/figure_data. (TIFF) [file pbio.1002452.s034.tiff]

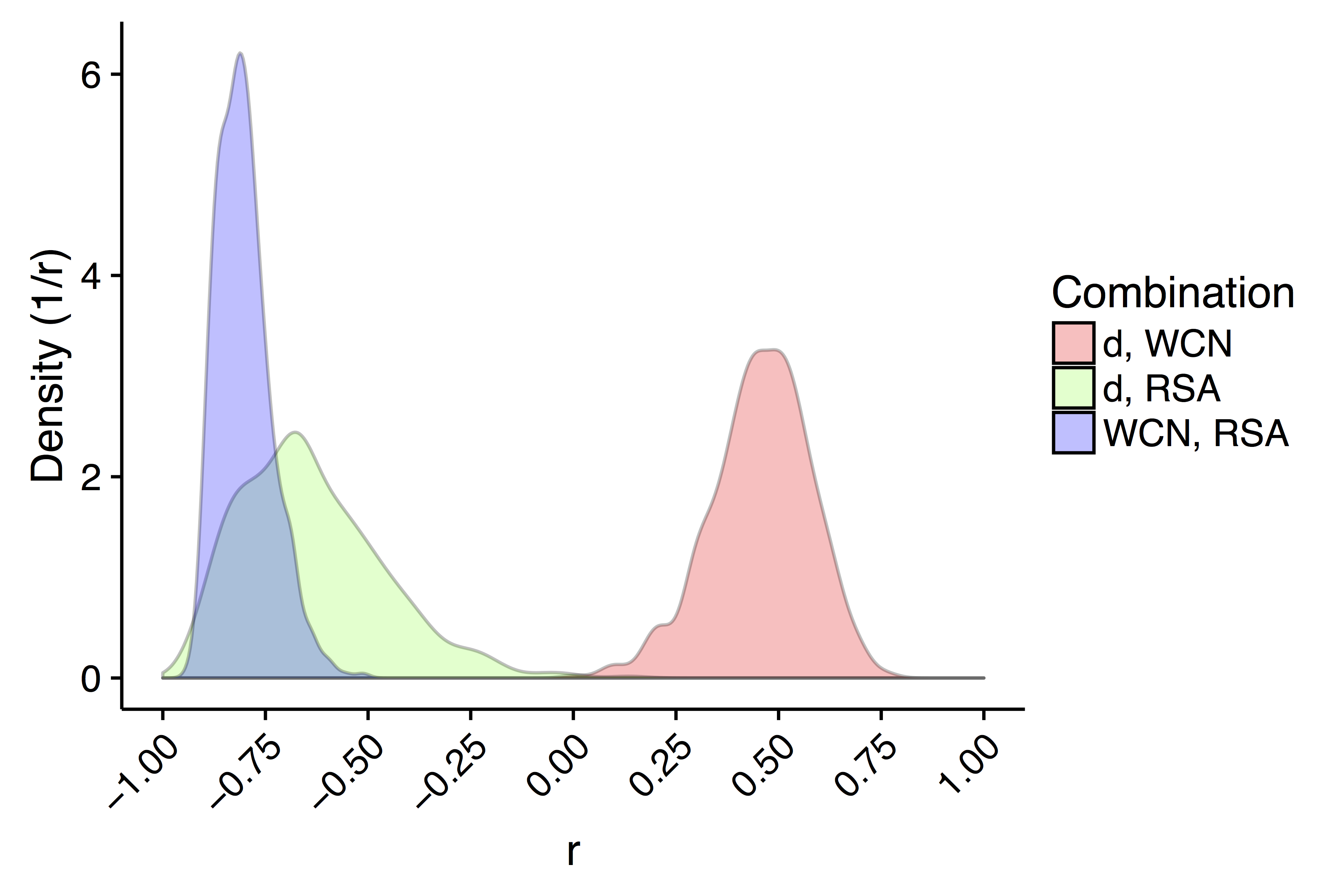

Supplement: S32 Fig — As in S2 Fig, but using biological assemblies with interface residues removed. Data underlying this figure are available on Github: https://github.com/benjaminjack/enzyme_distance/tree/master/figure_data. (TIFF) [file pbio.1002452.s035.tiff]

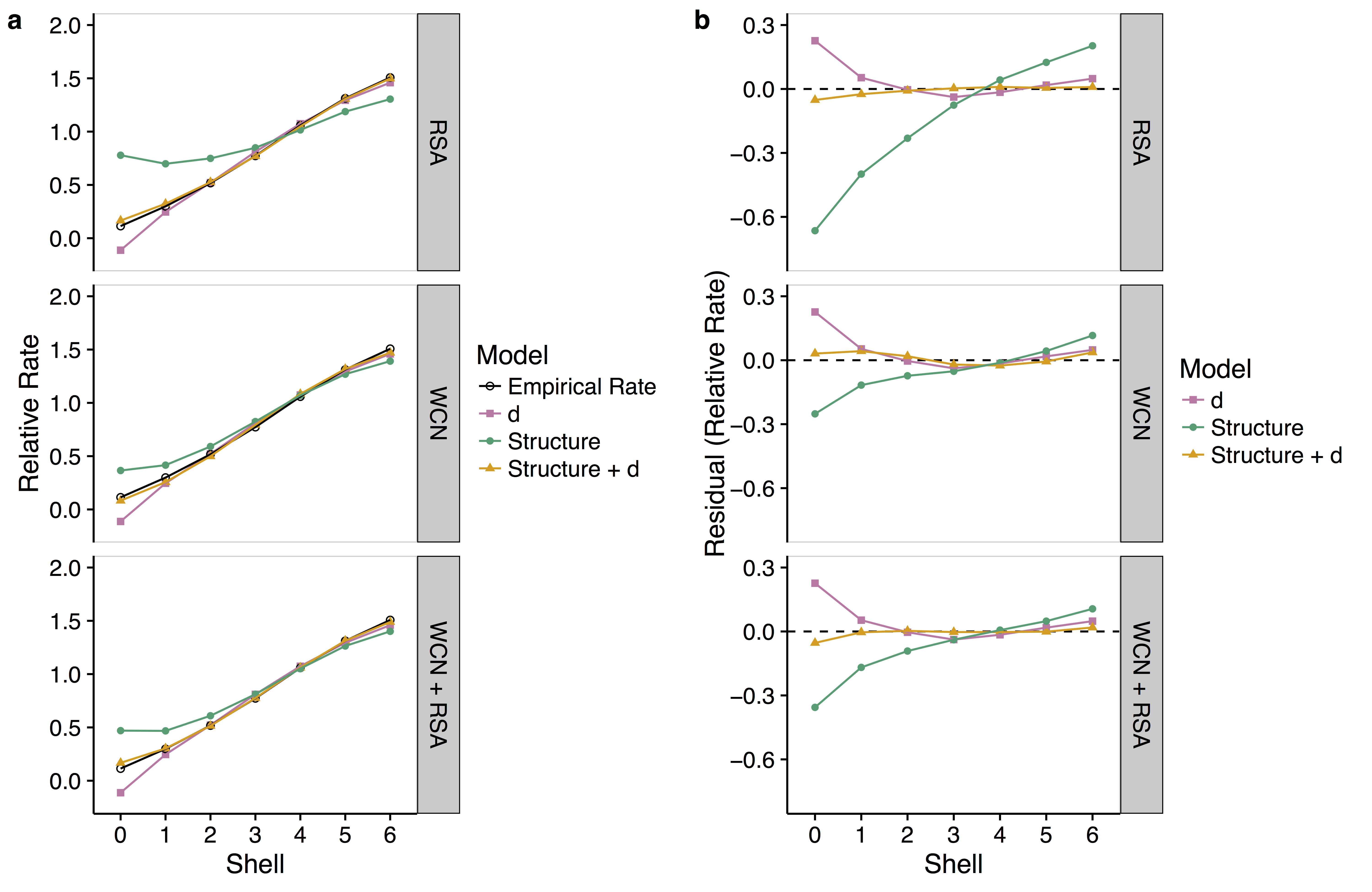

Supplement: S33 Fig — As in S3 Fig, but using biological assemblies with interface residues removed. Data underlying this figure are available on Github: https://github.com/benjaminjack/enzyme_distance/tree/master/figure_data/. (TIFF) [file pbio.1002452.s036.tiff]

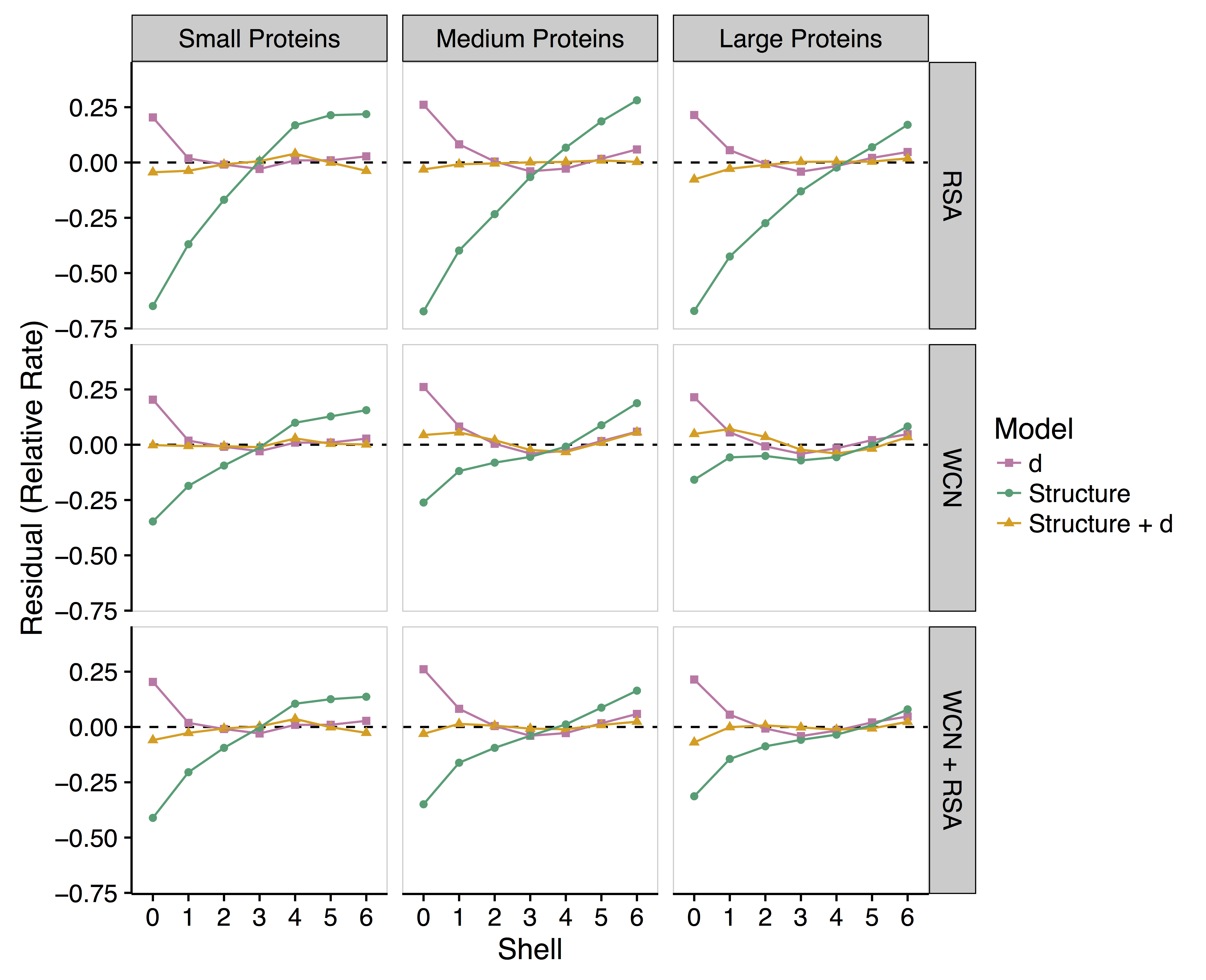

Supplement: S34 Fig — As in S5 Fig, but using biological assemblies with interface residues removed. Data underlying this figure are available on Github: https://github.com/benjaminjack/enzyme_distance/tree/master/figure_data/. (TIFF) [file pbio.1002452.s037.tiff]
