# Supplementary material for: Functional Sites Induce Long-Range Evolutionary Constraints in Enzymes
Source: PLoS Biol. 2016 May 3;14(5):e1002452. doi: 10.1371/journal.pbio.1002452 (PMC4854464; doi:10.1371/journal.pbio.1002452)
Supplement: S1 Table — The “Distance” column refers to the distance between the predicted catalytic residue and a true catalytic residue. “Optimized d” refers to selecting a reference residue that yields the maximum R2 between the set of distances to that residue and rates. This reference residue is predicted to be a catalytic residue. “Max. WCN” refers to a method in which the site with the maximum weighted contact number is assumed to be a catalytic residue. Evolutionary rate gradients recover active sites more often than does the site with maximum packing density (oddsratio = 2.8, p < 1.7 x 10^−15^, Fisher’s Exact Test). (DOCX) [file pbio.1002452.s038.docx]

**S1 Table**

|  | Model | |
| --- | --- | --- |
| Distance | Optimized *d* | Max. WCN |
| ≤ 7.5 Å | 290 | 162 |
| > 7.5 Å | 234 | 362 |
